# Dyskinetic crisis in *GNAO1*-related disorders: clinical perspectives and management strategies

**DOI:** 10.3389/fneur.2024.1403815

**Published:** 2024-06-06

**Authors:** Jana Domínguez Carral, Carola Reinhard, Darius Ebrahimi-Fakhari, Nathalie Dorison, Serena Galosi, Giacomo Garone, Masa Malenica, Claudia Ravelli, Esra Serdaroglu, Laura A. van de Pol, Anne Koy, Vincenzo Leuzzi, Agathe Roubertie, Jean-Pierre Lin, Diane Doummar, Laura Cif, Juan Darío Ortigoza-Escobar

**Affiliations:** ^1^Member of the ERN EpiCARE, Epilepsy Unit, Department of Child Neurology, Institut de Recerca Sant Joan de Déu, Barcelona, Spain; ^2^Centre for Rare Diseases and Institute of Medical Genetics and Applied Genomics, University Hospital Tübingen, Tübingen, Germany; ^3^European Reference Network for Rare Neurological Diseases (ERN-RND), Tübingen, Germany; ^4^Movement Disorders Program, Department of Neurology, Boston Children's Hospital, Harvard Medical School, Boston, MA, United States; ^5^Dyspa Unit, Pediatric Neurosurgery, Hôpital Fondation Rothschild, Paris, France; ^6^Department of Human Neuroscience, Sapienza University of Rome, Rome, Italy; ^7^Neurology, Epilepsy and Movement Disorders Unit, IRCCS Bambino Gesù Children Hospital, Rome, Italy; ^8^Department of Neuroscience, Mental Health and Sensory Organs (NESMOS), Faculty of Medicine and Psychology, Sapienza University of Rome, Rome, Italy; ^9^Member of the ERN EpiCARE, Department of Pediatrics, University Hospital Center Sestre Milosrdnice, Zagreb, Croatia; ^10^Sorbonne Université, Service de Neuropédiatrie-Pathologie du développement, Centre de référence neurogénétique, Hôpital Trousseau AP-HP.SU, Paris, France; ^11^Department of Pediatric Neurology, Gazi University Faculty of Medicine, Ankara, Türkiye; ^12^Emma Children’s Hospital, Amsterdam Universitary Medical Centers, Amsterdam, Netherlands; ^13^Department of Child Neurology, Amsterdam Universitary Medical Centers, Vrije Universiteit, Amsterdam, Netherlands; ^14^Department of Pediatrics, Faculty of Medicine and University Hospital Cologne, University of Cologne, Cologne, Germany; ^15^CHU Montpellier, Département de Neuropédiatrie, INM, Université de Montpellier, Inserm U, Montpellier, France; ^16^Children's Neurosciences Department, Evelina London Children's Hospital, Guy's and St Thomas' NHS Foundation Trust, London, United Kingdom; ^17^Women and Children's Institute, Faculty of Life Sciences and Medicine (FolSM), King's College London, London, United Kingdom; ^18^Département de Neurochirurgie, Unité des Pathologies Cérébrales Résistantes, Hôpital Gui de Chauliac, Centre Hospitalier Universitaire de Montpellier, Montpellier, France; ^19^Service de Neurologie, Department of Clinical Neurosciences, Lausanne University Hospital (CHUV), Lausanne, Switzerland; ^20^Laboratoire de Recherche en Neurosciences Cliniques, Montferrier-sur-Lez, France; ^21^Movement Disorders Unit, Department of Child Neurology, Institut de Recerca Sant Joan de Déu, Barcelona, Spain; ^22^U-703 Center for Biomedical Research on Rare Diseases (CIBER-ER), Instituto de Salud Carlos III, Barcelona, Spain

**Keywords:** *GNAO1*, dyskinetic crisis, movement disorders, deep brain stimulation, dystonia

## Abstract

**Background:**

*GNAO1*-related disorders (*GNAO1*-RD) encompass a diverse spectrum of neurodevelopmental and movement disorders arising from variants in the *GNAO1* gene. Dyskinetic crises, marked by sudden and intense exacerbations of abnormal involuntary movements, present a significant challenge in *GNAO1*-RD.

**Objectives:**

This study aimed to establish a standardized framework for understanding dyskinetic crises, addressing crucial aspects such as definition, triggers, diagnostic criteria, complications, and management strategies.

**Methods:**

A Delphi consensus process was conducted involving international experts in *GNAO1*-RD. The panel of thirteen experts participated in three voting rounds, discussing 90 statements generated through a literature review and clinical expertise.

**Results:**

Consensus was achieved on 31 statements, defining dyskinetic crises as abrupt, paroxysmal episodes involving distinct abnormal movements in multiple body regions, triggered by emotional stress or infections. Dyskinetic crises may lead to functional impairment and complications, emphasizing the need for prompt recognition. While individualized pharmacological recommendations were not provided, benzodiazepines and clonidine were suggested for acute crisis management. Chronic treatment options included tetrabenazine, benzodiazepines, gabapentin, and clonidine. Deep brain stimulation should be considered early in the treatment of refractory or prolonged dyskinetic crisis.

**Conclusion:**

This consensus provides a foundation for understanding and managing dyskinetic crises in *GNAO1*-RD for clinicians, caregivers, and researchers. The study emphasizes the importance of targeted parental and caregiver education, which enables early recognition and intervention, thereby potentially minimizing both short- and long-term complications. Future research should concentrate on differentiating dyskinetic crises from other neurological events and investigating potential risk factors that influence their occurrence and nature. The proposed standardized framework improves clinical management, stakeholder communication, and future *GNAO1*-RD research.

## Introduction

*GNAO1*-related disorder (*GNAO1*-RD) is a rare neurodevelopmental and movement disorder caused by pathogenic variants in the *GNAO1* gene (1), encoding a G protein subunit crucial for neuronal signaling. *GNAO1*-RD exhibits significant clinical heterogeneity, with core symptoms consisting of early-onset epilepsy, developmental delay/intellectual disability, and a hyperkinetic movement disorder, which typically includes chorea, dystonia, and myoclonus (2). A particularly challenging and poorly understood aspect involves recurrent episodes of acute exacerbations of hyperkinetic movement disorders, which have been termed dyskinetic crises.

Despite their clinical significance, dyskinetic crises have not been well defined, which has hindered effective communication among healthcare professionals and impeded research efforts. Recently, the term “*dyskinetic crisis*” was defined by Dominguez-Carral and colleagues (2) as “*sudden and marked exacerbation of abnormal involuntary movements (dyskinesias), which are distinct in onset and duration from the baseline dyskinetic movements of the patient, such as dystonia, chorea, or athetosis. During a dyskinetic crisis, alterations in facial expression may manifest, which are different from epileptic seizures, as there is no loss of awareness or disconnection from the surrounding environment* (2).”

Dyskinetic crises may have significant effects on the quality of life of both patients and their caregivers. It is essential to be aware of the potential short- and long-term complications of these crises, such as rhabdomyolysis (3–5), long bone fractures (6, 7) and renal failure (4, 8, 9), as well as complications like denutrition (3, 10), hyperthermia (3, 11), and respiratory distress (10, 12, 13). In order to address dyskinetic crises, prompt intervention and tailored management strategies are essential. In severe cases, patients may need extended ICU stays, possibly requiring a tracheostomy (10, 11), or gastrostomy (10, 12). There are documented cases of dyskinetic crises resulting in fatalities (6, 7, 12, 14), highlighting the urgent need for effective interventions. Considering the significant morbidity and mortality, dyskinetic crises were added as a severity indicator to the *GNAO1*-RD severity score (2).

The pathophysiology of dyskinetic crises is complex and not fully understood. In the mammalian striatum, Gαo plays a pivotal role in controlling movement in D1 and D2 dopamine receptor-expressing medium spiny neurons. *GNAO1* pathogenic variants, including G42R, G203R, and R209C, are associated with loss of function and dominant negative effects, which have an adverse effect on motor behaviors and contribute to the development of dyskinetic crisis (15). The pathogenic variants cause disturbances in cAMP signaling pathways (16), which impede the regulation of GABA-B and α2 receptors and subsequently impact the release of neurotransmitters (9). Furthermore, these pathogenic variants affect functional polarity in developing neurons, calcium signaling, and neurite outgrowth by interfering with cytoskeletal remodeling and neuronal firing (17).

To address dyskinetic crises in *GNAO1*-RD, we have convened a Delphi consensus review involving an international panel of experts. Consensus was reached on a number of aspects pertaining to dyskinetic crises, including their definition, triggers, diagnostic criteria, potential short- and long-term complications, and effective management strategies and interventions. The establishment of this standardized framework for understanding dyskinetic crises will improve clinical management and future research in the field of *GNAO1*-RD.

## Materials and methods

**Figure 1:**
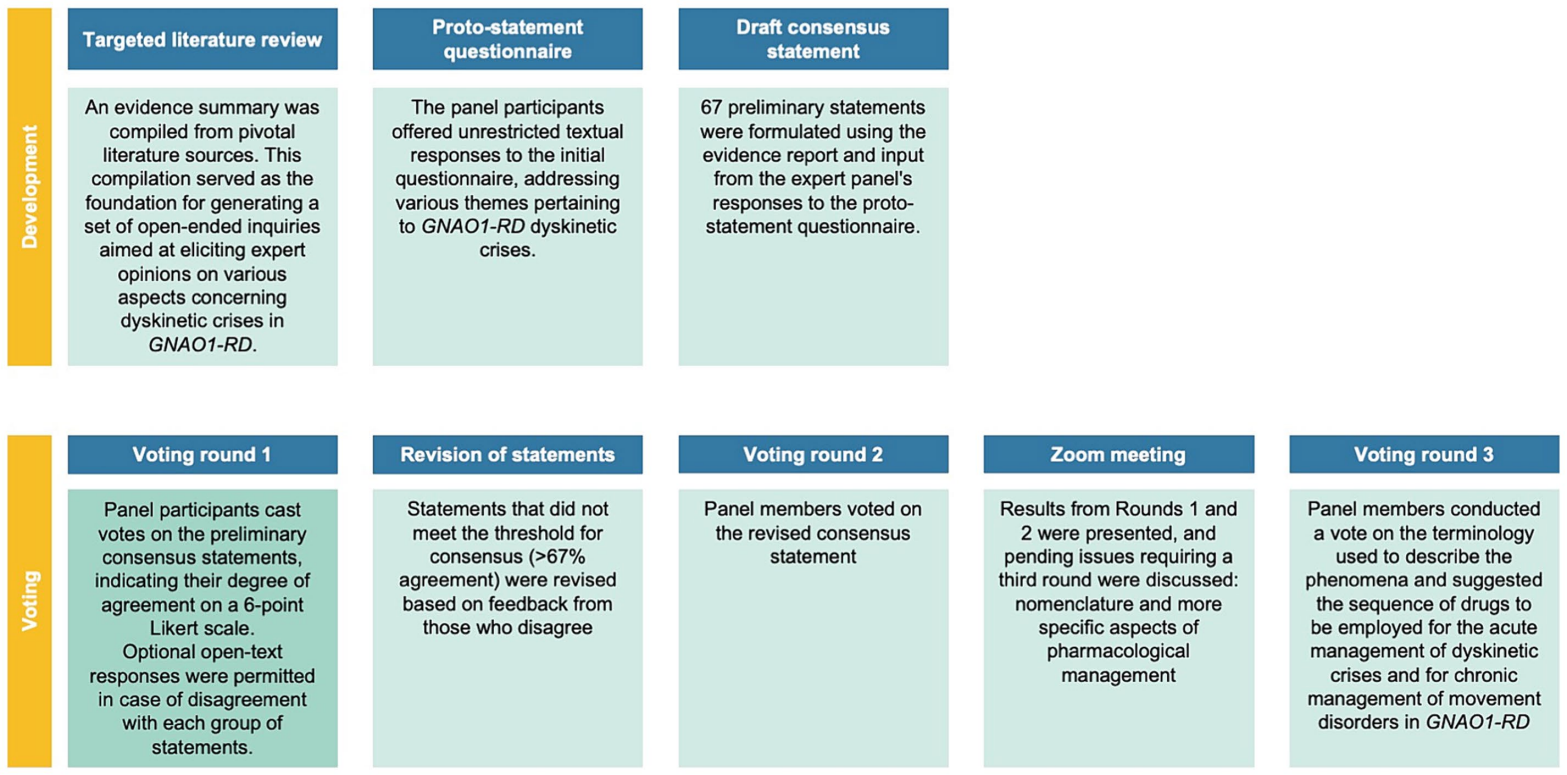
Overview of the *GNAO1*-RD dyskinetic crisis consensus process.

**Figure 2:**
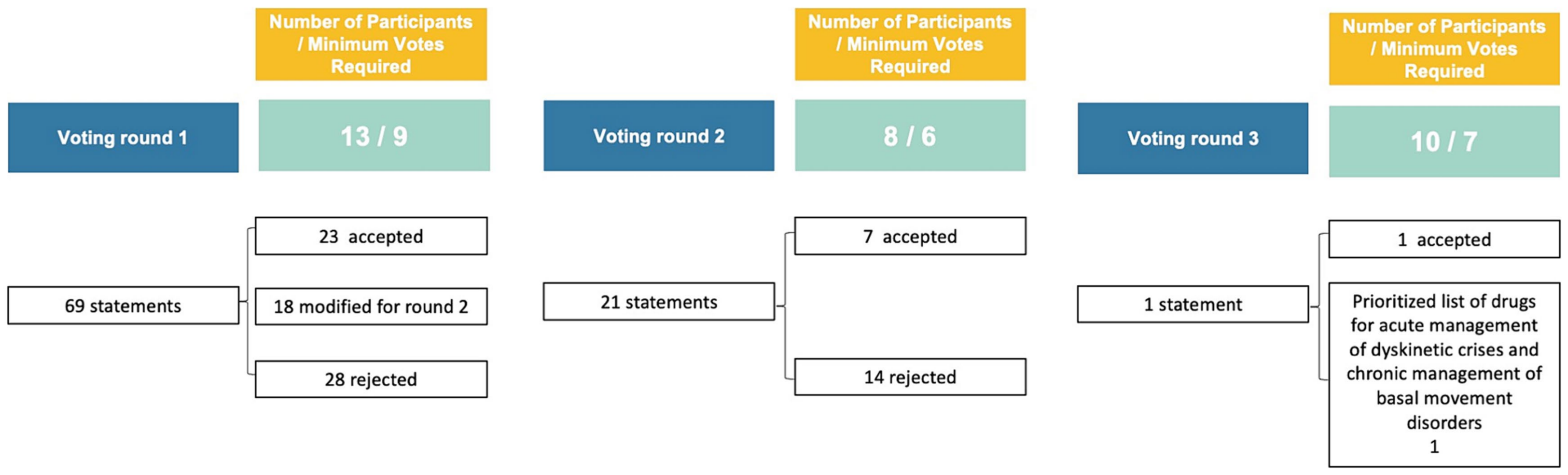
Participant numbers, minimum votes required, and statements for rounds 1, 2, and 3.

In this study, a Delphi consensus process was conducted to gather expert opinions on various aspects related to *GNAO1*-RD dyskinetic crises. A steering committee (Supplementary Table S1), along with 13 international experts, was involved in the process. The study involved multiple voting rounds, where experts anonymously voted on statements using a 6-point Likert scale. The predefined consensus threshold was set at ≥67%, and statements not reaching this threshold were revised based on feedback. Discussions during the process led to the emergence of new topics, including appropriate terminology and medical management strategies, which were also voted on in the third round. Further details on the methodology, literature review, and statements can be found in the Supplementary Data and Supplementary Figure S1. Figures 1, 2 provide an overview of the *GNAO1*-RD dyskinetic crisis consensus process. Patients included in the videos were selected from the clinical practice of the authors, and their participation was contingent upon obtaining informed consent for the publication of these videos.

## Results

### Overview

Thirty-five articles were identified in a targeted literature review (Supplementary Data; Supplementary Figure S1). Based on this evidence, 69 draft statements were developed. Following the participants’ responses in the initial round, 18 statements were revised for the subsequent round, and an additional 3 statements were formulated for the second round. Following the investigator meeting at this stage, a statement was formulated regarding the nomenclature of movement disorder phenomena, along with a prioritized list of drugs for acute pharmacological management of dyskinetic crises and chronic management of background movement disorders.

### Literature review

In the literature, 102 *GNAO1*-RD patients with episodes consistent with dyskinetic crises have been described. The data extracted from the literature review are presented in Supplementary Data. It is important to reference the various terms used in the literature to describe this movement disorder phenomenon: continuous, generalized involuntary movements (3), exacerbation of dyskinesia (18, 19), acute exacerbation (9, 20), episodes of dyskinetic movement (8, 21), severe episodes of paroxysmal choreoathetosis (12), worsening of extrapyramidal symptomatology (22), dyskinetic episodes (13), paroxysmal episodes (13), chorea episodes (23), movement disorder fluctuations (24, 25), recurrent episodes of hyperkinesia (26), intermittent hyperkinesia (27), episodic deterioration of the movement disorders (28), worsening of hyperkinetic movement (29), hyperkinetic crisis (26, 30), dyskinetic crisis (2), dystonic-dyskinetic movements (31), and spells (6). In its most severe manifestation, this motor phenomenon has been referred to as dyskinetic status (13, 24, 32), hyperkinetic state (26), status dystonicus (10, 33, 34), dystonic storm (4, 5), intractable dystonia (35), or a movement disorder emergency (13).

In the Supplementary Data, the genotype of patients, triggers, acute pharmacological management, chronic management, and deep brain stimulation, as well as complications, are described in detail. Supplementary Figure S2 shows the genotype of the 102 *GNAO1*-RD reported cases with dyskinetic crises.

### Voting participation and consensus

All 13 voting members of the Delphi panel participated in at least one round of the process and thus qualified for membership in the final Delphi panel. In round 1, the participation rate was 100%. After this voting round, 23 of the 69 statements reached consensus. 28 statements failed to reach consensus; these statements were revised based on feedback. In round 2, eight panel members voted on the revised statements. During this voting round, seven additional statements reached consensus. The third voting round focused on refining the terminology used to describe the phenomena and delving into more detailed aspects of medical management. The participation rate for the third round was 77% (10/13). Not all experts were able to participate in all three rounds.

### Consensus statements

In total, the Delphi panel agreed on 31 consensus statements. The statements and the detailed voting responses are summarized, by theme, in Supplementary Tables S2–S13.

#### Dyskinetic crisis: definition

##### Statements 1–7

**Figure 3:**
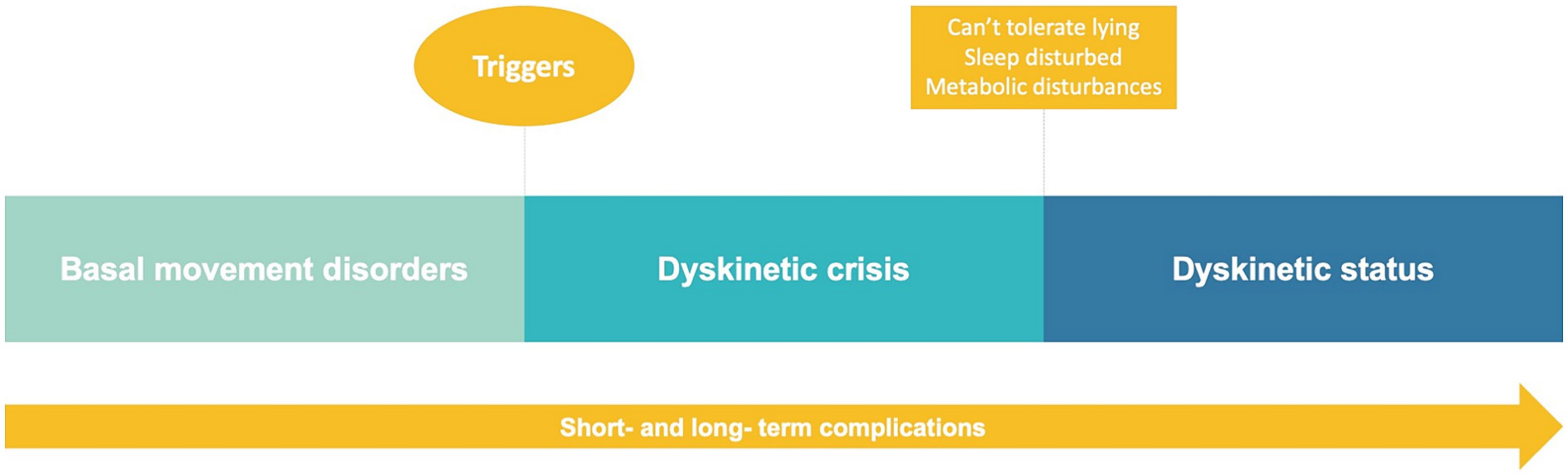
Diagram illustrating movement disorders in *GNAO1*-RD.

A dyskinetic crisis in *GNAO1*-RD is characterized by abrupt, paroxysmal episodes of abnormal involuntary movements (Figure 3). These episodes typically involve multiple body regions, including the upper and lower limbs, trunk, and face. Dyskinetic crises in *GNAO1*-RD are commonly associated with dystonia, choreoathetosis, ballismus, or a combination of these movement disorders. They can last for minutes to hours (less commonly days to weeks) and may occur spontaneously or be triggered by various factors. Individuals experiencing a dyskinetic crisis often face significant functional impairment and a loss of voluntary control over their movements. These crises can recur multiple times per day. Additionally, it is important to note that in *GNAO1*-RD patients, the term “dyskinetic status” should be used instead of “dystonic status.” However, it is crucial to recognize that both terms, dystonic status and dyskinetic status, are often interchangeable, particularly in the context of *GNAO1*-RD. The choice between “dystonic” or “dyskinetic” may depend more on the predominant phenomenology observed in each individual case, whether it manifests as dystonia or choreoathetosis. In the third round, the term “dyskinetic crisis” was endorsed to characterize this movement disorder phenomenon (Supplementary Videos S1–S4).

##### Comments

A dyskinetic crisis encompasses any sudden alteration in the baseline movement disorder, regardless of its duration or severity. This implies that previously used terms such as “spells” or “episodes” should be discouraged and replaced. The involuntary movements observed during a dyskinetic crisis may or may not deviate from the baseline motor patterns of the patient. These movements could resemble their usual movement disorders (most often chorea and dystonia), but with increased intensity or severity. Alternatively, they might manifest as entirely new movement disorders, such as ballism. Isolated orolingual dyskinesias should not be classified as dyskinetic crises, nor should any focal movement disorder (chorea or dystonia). Moreover, a focal movement should raise suspicion of a focally aware or impaired-aware epileptic seizure. The assessment of consciousness in *GNAO1*-RD dyskinetic crises presents a clinical challenge, as there is a belief that consciousness remains unimpaired during these events, although distinguishing this from seizures can be challenging and may require confirmation via EEG. However, it is crucial to note that reaching definitive conclusions about impaired consciousness or awareness in individuals with *GNAO1*-RD during dyskinetic crises necessitates additional research and evidence. Further studies are required to provide a more comprehensive understanding and clarification of this particular aspect of the condition. The diagnosis of *status dystonicus* or *dyskinetic status* will be made following the recommendations of Allen et al., which include patients presenting with intolerance to lying down and disturbed sleep, along with metabolic disturbances such as fever (not related to infection), dehydration, abnormal electrolytes, CK >1,000 IU/L, and myoglobinuria (36) (Figure 3). It is therefore important to differentiate dyskinetic crises from dystonic or dyskinetic status, which must meet the previously described criteria.

#### Dyskinetic crisis: clinical features aside from MD

##### Statements 13–15, 87

In *GNAO1*-RD, dyskinetic crises may coexist with autonomic symptoms such as diaphoresis, tachycardia, and blood pressure changes. Autonomic symptoms, in isolation, along with increased movements, do not by themselves define a dyskinetic crisis. Autonomic symptoms should be recognized as potential features of various movement disorder presentations, not exclusive to dyskinetic crises, warranting a comprehensive evaluation. Speech and swallowing can also be affected by dyskinetic crises. Patients frequently experience fatigue or weakness following a dyskinetic crisis.

#### Dyskinetic crisis: triggers or precipitant factors

##### Statements 16–18, 83

Dyskinetic crises in individuals with *GNAO1*-RD are commonly precipitated by stressors such as emotional stress, anxiety, excitement, and pain, as well as infections including respiratory or urinary tract infections, underscoring the importance of recognizing and avoiding these triggers when devising treatment strategies.

##### Comments

Certain triggers described anecdotally in the literature lack substantial empirical experience. Consequently, a consensus could not be reached on specific triggers previously mentioned, including bowel movements (6), high ambient temperature (12, 28), purposeful movements (12, 13, 26), sound (14), and menstruation (23). Dyskinetic crises can occasionally arise in the absence of identifiable precipitating factors; therefore, it is indispensable that patients and families be appropriately informed.

#### Dyskinetic crisis: differences compared to background movement disorders

A consensus could not be achieved in this area.

##### Comments

Dyskinetic crises can resemble the patient’s baseline movement disorder. Autonomic symptoms may be observed in other movement disorders without being specific to dyskinetic crises.

#### Dyskinetic crisis: distinctive patterns or variations

A consensus could not be achieved in this area.

##### Comments

Further evidence is essential to determine whether more severe phenotypes of *GNAO1*-RD correlate with a higher incidence of dyskinetic crises. Notably, some patients with milder phenotypes, characterized by a more dystonic presentation, occasionally limited to isolated cervical dystonia, have never experienced dyskinetic crises (20, 37). It is crucial to highlight that individuals with *GNAO1*-RD might encounter dyskinetic crises several years after the onset of dystonia. Additionally, comprehensive research is required to investigate potential variations in the duration, frequency, and severity of dyskinetic crises across different age groups. Similarly, there is a need to explore whether the incidence of dyskinetic crises diminishes with age, necessitating further investigation. Natural history studies are imperative to assess whether the characteristics of crises change within the same patient over time. This includes examining potential genotype correlations with dyskinetic crises as well as exploring whether accompanying neurological features, such as cognitive impairment, epilepsy, or developmental delay, influence the nature of the dyskinetic crisis.

#### Dyskinetic crisis: diagnosis criteria and guidelines

##### Statements 39–44

In the context of *GNAO1*-RD, diagnosing dyskinetic crises is challenging due to the absence of specific criteria. Individuals with acute, paroxysmal, and involuntary dyskinetic movements should be considered for dyskinetic crisis evaluation. Video documentation of these episodes is valuable, offering visual insights into the frequency and characteristics of abnormal movements. Longitudinal observation and comprehensive clinical assessments aid in identifying diagnostic patterns. Unlike epileptic seizures, dyskinetic crises most probably preserve awareness and exhibit inconsistent bilateral movements. Therefore, differential diagnosis involves excluding other potential causes of paroxysmal movement disturbances, emphasizing the need for meticulous evaluation and monitoring in individuals presenting with such symptoms in the *GNAO1*-RD context.

##### Comments

In the context of differentiating dyskinetic crises from other neurological events such as epileptic seizures, clarification is needed regarding the categorization of these events. Obtaining EEG recordings from patients experiencing dyskinetic crises poses significant challenges. While EEG recordings can be valuable, they are not obligatory; the distinctive patterns and accompanying signs observed during dyskinetic crises are likely adequate for distinguishing them from epileptic seizures on clinical grounds.

#### Dyskinetic crisis: potential short- and long-term complications

##### Statements 46, 88, 89

Dyskinetic crisis can interfere with activities of daily living and functional independence, resulting in a lack of autonomy and decreased quality of life for individuals with *GNAO1*-RD. Dyskinetic crisis may disrupt the ability to maintain oral feeding and should be considered as a factor affecting nutritional management. Dyskinetic crisis may be associated with an increased risk of falls and/or injuries (pain, tongue biting, joint dislocations, bone fractures, etc.).

##### Comments

Differentiating between the motor deterioration resulting from frequent dyskinetic crises and the putative neurodegeneration associated with *GNAO1*-RD proves to be a challenging task. The distinction between these two factors is intricate and often unclear, making it difficult to ascertain the precise cause of the motor decline in individuals affected by *GNAO1*-RD. The assessment of motor decline in such cases is further complicated by the immediate harm and injuries patients experience during these events. In the literature, complications such as pressure ulcers (6), superficial injuries (7, 9), skin breakdown (7), femur (6) or bone fractures (7), obstinate constipation (13), acute colitis (4), pneumatosis hepatis (4), and anxiety (25) have been described.

#### Dyskinetic crisis: management strategies and interventions

##### Statements 49–55

**Figure 4:**
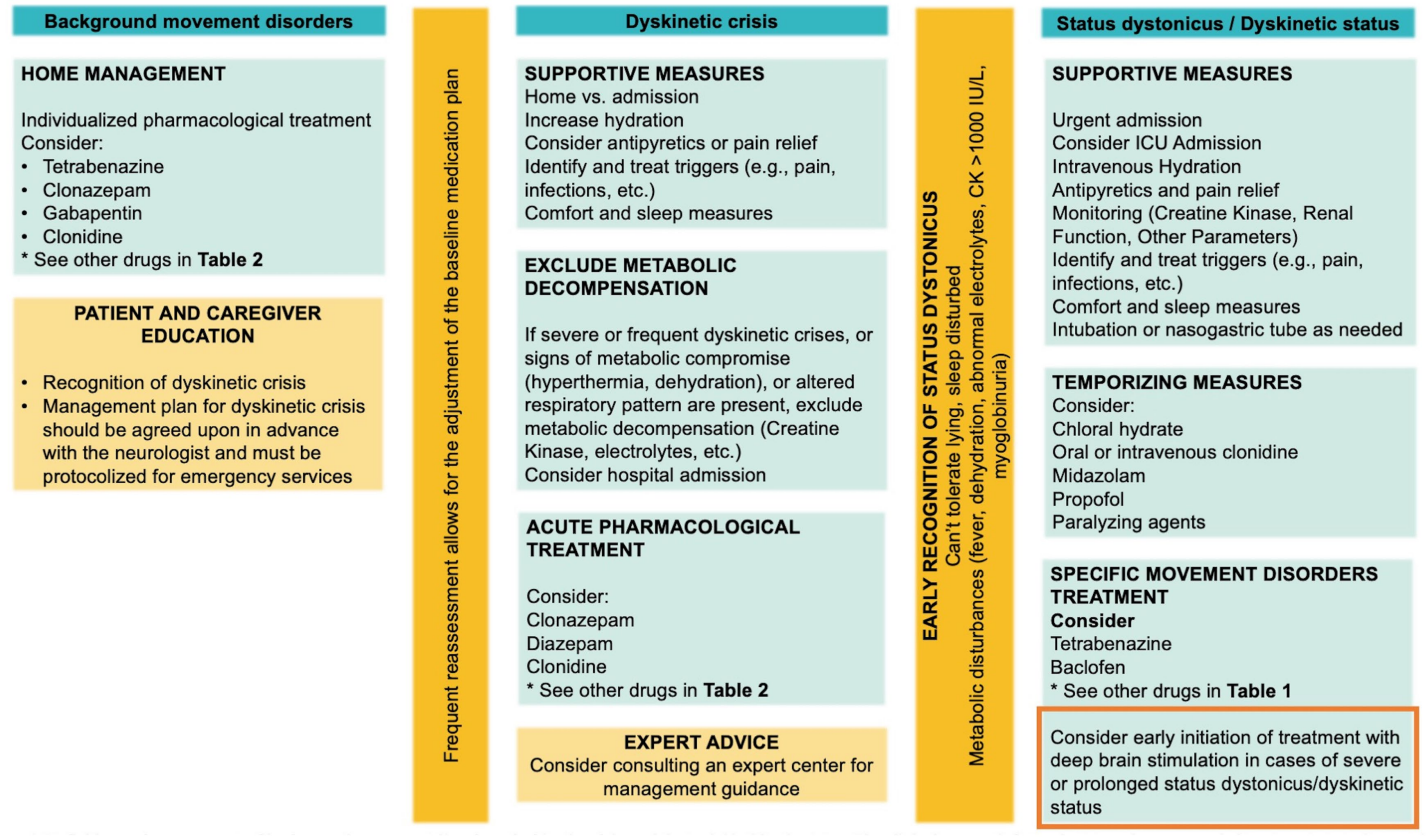
Management of background movement disorders, dyskinetic crisis, and dystonic/dyskinetic status. The clinical approach for each state and recommended treatment strategies are shown. Importantly, consider the early initiation of treatment with DBS.

In managing dyskinetic crises in *GNAO1*-RD, patients should receive comprehensive care following the guidelines outlined in the Dystonia Severity Action Plan (DSAP) (36), which includes criteria for escalation of care level, as needed medications, and appropriate laboratory tests. During prolonged dyskinetic crises, healthcare providers must rule out complications such as rhabdomyolysis, electrolyte imbalances, and renal abnormalities. Optimal management involves continuous monitoring and adjustment of medication regimens based on clinical response and side effect profiles. Deep brain stimulation (DBS) has emerged as a promising therapeutic approach, particularly for severe and refractory dyskinetic crises, significantly ameliorating motor symptoms. For patients experiencing frequent or severe crises requiring hospitalization, early consideration of DBS in the treatment plan is crucial. Additionally, educating and supporting parents and caregivers is essential, as is fostering a coordinated approach and ensuring adherence to treatment plans. A personalized strategy that integrates various therapeutic modalities, including medications, surgical options (DBS), and assistive devices, has proven effective in addressing dyskinetic crises in *GNAO1*-RD, highlighting the significance of tailored, multidisciplinary interventions. Figure 4 summarizes the recommended management of background movement disorders, dyskinetic crisis, and dystonic/dyskinetic status.

##### Comments

Additionally, employing clonidine rescue therapy in combination with chloral hydrate (where available) has proven to be beneficial in preemptively addressing *GNAO1*-RD dyskinetic crises, both in a home and hospital setting, effectively averting respiratory compromise (9). It is important to note that chloral hydrate is not available in the US, Italy, or France. Furthermore, considering the use of dexmedetomidine as an alternative in intermediate care and ICU settings, where IV clonidine may not be feasible. In certain cases, plasma exchange (3) or dialysis (13) have been employed in the treatment of complications, including rhabdomyolysis.

#### Dyskinetic crisis: acute pharmacological and surgical approach

Individualized pharmacological recommendations for the acute treatment of dyskinetic crises cannot be provided. Experts commonly preferred clonazepam, diazepam, and clonidine as the primary medications for managing dyskinetic crises, as indicated in Table 1, which presents the list of drugs ranked by first, second, or third preference. It is crucial to reiterate that chloral hydrate is not readily obtainable in many countries, including the United States, France, and Italy.

**Table 1:** Order of drug selection for the acute management of dyskinetic crisis and basal movement disorder in *GNAO1*-RD.

Acute treatment for dyskinetic crisis								
1	2	3	4	5	6	7	8	9
Clonazepam	Clonazepam	Diazepam	Clonazepam	Clonazepam	Clonazepam	Tetrabenazine	Diazepam	Clonidine
Diazepam	Diazepam	Clonazepam	Diazepam	Clonidine	Diazepam	Clonidine	Clonazepam	Clonazepam
Clonidine	Clonidine	Chloral hydrate	Chloral hydrate	Diazepam	Clonidine	Trihexyphenidyl	Chloral hydrate	Carbamazepine
Chloral hydrate	Carbamazepine	Clonidine	Clonidine	Chloral hydrate	Chloral hydrate	Amitriptyline	Clonidine	Gabapentin
Baclofen	Gabapentin	Gabapentin	Baclofen	Gabapentin	Gabapentin	Clonidine	Baclofen	Baclofen
Carbamazepine	Topiramate	Baclofen	Gabapentin	Carbamazepine	Baclofen	Clonazepam	Gabapentin	Diazepam
Topiramate	Baclofen	Carbamazepine	Carbamazepine	Topiramate	Carbamazepine	Diazepam	Carbamazepine	Topiramate
Midazolam	Chloral hydrate	Topiramate	Topiramate	Baclofen	Topiramate	Baclofen	Topiramate	Chloral hydrate
Tetrabenazine		Midazolam			Midazolam	Carbamazepine		
						Gabapentin		
						Topiramate		
						Chloral hydrate		
Chronic treatment for basal movement disorders								
1	2	3	4	5	6	7	8	9
Tetrabenazine	Clonazepam	Tetrabenazine	Tetrabenazine	Tetrabenazine	Trihexyphenidyl	Trihexyphenidyl	Clonazepam	Clonazepam
Clonazepam	Gabapentin	Clonidine	Trihexyphenidyl	Clonazepam	Lorazepam	Tetrabenazine	Gabapentin	Diazepam
Diazepam	Tetrabenazine	Clonazepam	Gabapentin	Gabapentin	Gabapentin	Clonidine	Clonidine	Gabapentin
Clonidine	Trihexyphenidyl	Gabapentin	Clonazepam	Baclofen	Clonazepam	Clonazepam	Baclofen	Carbamazepine
Chloral hydrate	Clonidine	Diazepam	Diazepam	Diazepam	Clonidine	Topiramate	Chloral hydrate	Clonidine
Baclofen	Carbamazepine	Baclofen	Baclofen	Carbamazepine	Baclofen	Baclofen	Diazepam	Chloral hydrate
Carbamazepine	Topiramate	Carbamazepine	Clonidine	Clonidine	Chloral hydrate	Gabapentin	Carbamazepine	Baclofen
Gabapentin	Diazepam	Chloral hydrate	Chloral hydrate	Topiramate	Diazepam	Diazepam	Topiramate	Topiramate
Topiramate	Baclofen	Topiramate	Carbamazepine	Chloral hydrate	Carbamazepine	Carbamazepine		
	Chloral hydrate		Topiramate		Topiramate	Chloral hydrate		
First choice								
Second choice								
Third choice								

Each number represents the opinion of one of the participating experts.

##### Comments

The selection of drugs by the experts did not include general anesthetics, paralytics, opioids, or other medications described in the literature, such as dexmedetomidine (5, 6), propofol (6, 22, 28, 29, 31), pentobarbital (6), thiopental (31), phenobarbital (6, 13, 22, 23, 28, 30, 38), phenytoin (13), ketamine (5, 8), vecuronium (6), dantrolene (3), tiapride (22, 23, 38), triclofos sodium (3), morphine (4), hydromorphone (4), fentanyl (6, 11, 28, 29), which are commonly used in intensive care settings. Additionally, drugs used anecdotally in some reported cases, such as trazodone (6), biperiden (8), chlorpromazine (8), bethanechol (6), tizanidine (30, 39), metamizole (8), acetazolamide (8, 11), propranolol (8), nitrazepam (24), cannabis (30), methylphenidate (20), amantadine (20), pramipexol (18), and benserazide (24), were not included. There are some cases reported in the literature with an excellent response to topiramate (23), oxcarbazepine (38), or gabapentin (3). In some cases, the ketogenic diet (8, 14, 39) has also been used to control dyskinetic crises without positive effects. Other drugs, such as corticosteroids (38) or immunoglobulins (38), lacking a clear mechanism of action related to dyskinetic crises, were also not included in the drug selection. In other anecdotal cases, neurosurgical procedures such as bilateral pallidotomy (6, 13) and intrathecal baclofen therapy (9, 24) have been performed, with partially effective outcomes. Supplementary Figure S3 shows the pharmacological and surgical interventions reported in the literature. Finally, the administration route should be chosen according to the crisis’ severity, bulbar region involvement possibly preventing safe swallowing, availability of a gastric tube, and possible coexistence of vomiting or ileus.

#### Basal movement disorder: chronic pharmacological approach

Individualized pharmacological recommendations for the chronic management of movement disorders in *GNAO1*-RD patients cannot be provided. Tetrabenazine, clonazepam, gabapentin, and clonidine emerged as the most preferred medications for managing movement disorders in *GNAO1*-RD patients. Table 1 presents the ranking of these drugs based on first, second, or third preference. It is worth noting that tetrabenazine has been prescribed to children younger than 12 months of age, with doses up to 10 mg/kg/day administered to *GNAO1*-RD patients under the care of experts in this field. It is important to note the potential role of DBS, although its application in emergency situations is inconsistent, despite its proven efficacy.

#### Dyskinetic crisis: additional factors and considerations

##### Statements 63–64

Upon *GNAO1*-RD diagnosis, initiating parental or caregiver education is imperative, encompassing detailed information about dyskinetic crises, their nature, and appropriate actions to take when a child experiences such episodes. Additionally, the variability in duration and frequency of dyskinetic crisis episodes among individuals with *GNAO1*-RD must be taken into account when characterizing the condition.

##### Comments

As time progresses, parents or primary caregivers often develop a keen ability to distinguish between these dyskinetic crises and epileptic seizures. Despite the initial difficulty, the caregivers’ increasing familiarity with the patient’s condition enables them to discern the distinct characteristics of dyskinetic crises, contributing to a more accurate identification and understanding of these events over time.

## Discussion

*GNAO1*-RD represents a range of neurodevelopmental and movement disorders arising from pathogenic variants in the *GNAO1* gene. Dyskinetic crises stand out as a highly distinctive feature, with significant morbidity and mortality. Using a modified Delphi consensus procedure and a literature review, we generated 90 statements regarding the dyskinetic crisis. These statements, which reflect the consensus of field experts, offer insights into the diagnostic and therapeutic landscapes of dyskinetic crises in patients with *GNAO1*-RD. Through this rigorous methodology, we reached a consensus on crucial aspects, including the definition of dyskinetic crises. This consensus framework addresses a substantial body of knowledge and management of various aspects of dyskinetic crises, yet it also reveals certain gaps in the clinical setting. By establishing a standardized foundation for research and clinical management of *GNAO1*-RD, it sheds light on areas that require further attention and development.

In the context of defining the movement disorder phenomenon, we assert that the terminology employed to accurately depict this manifestation should encompass essential attributes, including deviations from baseline movement disorders, duration, and intensity. Following the voting process during the third round of discussions, the decision was reached to retain the term “*dyskinetic crisis*,” among other options considered. The term “crisis” signifies a sudden and intense manifestation or deterioration of a disorder, making it a fitting descriptor for the discussed phenomenon. Moreover, from the perspective of families with *GNAO1*-RD patients, referring to these events as “*dyskinetic crisis*” rather than using terms like “exacerbation” or “episode” might simplify communication and enhance clarity and understanding.

We also considered whether focal symptoms, such as orolingual dyskinesias (5, 9, 11, 26), which are highly prevalent and sometimes incapacitating in patients with *GNAO1*-RD, should be included in the term “*dyskinetic crisis*.” Although they may satisfy the paroxysmal onset and termination criterion, we have chosen to describe them separately from dyskinetic crises. Notably, orolingual dyskinesias have preceded the dyskinetic crisis in some cases.

An integral part of this consensus is the understanding of the triggers of these crises. The fact that *GNAO1*-RD has less-defined triggers, some of which may be anecdotal. Others, such as temperature changes (12, 28), the use of antiemetics (e.g., metoclopramide), and anticholinergics (e.g., scopolamine, trihexyphenidyl) (26), are shared with other diseases characterized by paroxysmal phenomena. It is recommended that these medications be avoided or used with caution by these patients, beginning with very small doses and gradually increasing them as necessary.

Regarding diagnosis, we believe that clinical evaluation alone is adequate for diagnosing a dyskinetic crisis and that additional complementary assessments, such as video EEG, are not routinely required.

In our study, a genotype–phenotype correlation regarding the risk of dyskinetic crises could not be established. According to previously published articles, the most frequent pathogenic variants, including c.736G > A (11 patients), c.607G > A (7 patients), c.626G > A (6 patients), c.625C > T (5 patients), and c.709G > A (3 patients), are also associated with more frequent dyskinetic crises (13). Therefore, it cannot be ruled out that the occurrence of dyskinetic crises is related to the frequency of a variant rather than the specific susceptibility of a pathogenic variant to develop dyskinetic crises.

Dyskinetic crises pose significant risks to patients, both in the short and long term, as evidenced by the literature. Hence, mitigating these crises is paramount. While individualized recommendations may vary, we advocate for a multi-faceted approach to acute pharmacological intervention, emphasizing the use of clonidine, benzodiazepines, and chloral hydrate (where accessible). These agents have shown efficacy in mitigating acute dyskinetic crises. Moreover, to forestall exacerbations and recurrent crises, we advocate for sustained pharmacological management of the underlying movement disorder. Tetrabenazine, benzodiazepines, gabapentin, and clonidine have demonstrated utility in this regard, offering a spectrum of options tailored to individual patient needs.

In parallel, we acknowledge the potential of surgical interventions, particularly DBS, in refractory cases. While further research is needed to clearly establish precise indications and optimize its utilization as an advanced therapeutic modality, the literature provides ample examples of cases where DBS treatment has proven effective in managing treatment-resistant dystonic status and preventing recurrent crises. Therefore, we underscore the importance of continued research to elucidate the precise indications and optimize the utilization of DBS as an early and advanced therapeutic modality.

In terms of treatment, we have not discussed potential pharmacological interventions currently under investigation, such as the use of caffeine (40) and zinc acetate (41). Regarding caffeine, Di Rocco et al. demonstrated its ability to attenuate hyperkinetic behavior in mutated R209H and E246K *Caenorhabditis elegans*. Additionally, the use of istradefylline, a selective adenosine A2A receptor antagonist, indicated that caffeine operates via both adenosine receptor-dependent and receptor-independent mechanisms. Furthermore, caffeine has been shown to effectively reduce the frequency and duration of paroxysmal movement disorders in *ADCY5* (42). Moreover, it has exhibited improvements in baseline movement disorders and various other motor and non-motor symptoms, consistently resulting in an enhanced quality of life. Regarding zinc acetate, a single-patient treatment involving a 3-year-old with a common GNAO1 mutation c607G > A/p.Gly203Arg, administered oral 50 mg Zn2+ daily for 11 months, resulted in the cessation of daily dyskinetic crises. These authors have categorized pathogenic Gαo mutations into three classes based on their sensitivity to Zn^2+^, with mutations in groups II and III potentially benefiting from zinc acetate treatment (41).

In the realm of movement disorders, it is also important to consider the differential diagnosis of dyskinetic crises with paroxysmal dyskinesias. In this regard, it is relevant to mention patients with *GNB1* encephalopathy, in whom paroxysmal dyskinesias lasting for hours may be observed, usually triggered by fever and related to awakening (16). Additionally, episodes of dyskinesias in patients with *ADCY5* mutations are noteworthy, consisting of severe, sleep-disrupting movements occurring during stages N2 and N3 of sleep (42, 43). An important distinction in the case of *GNB1* is the later age of onset compared to *GNAO1*-RD patients. Regarding *ADCY5*, a significant difference lies in its very close association with sleep.

Fundamentally, our research highlights the critical need for targeted parental and caregiver education following the diagnosis of *GNAO1*-RD. Parents and caregivers can become frontline advocates for managing their child’s condition if they are well-informed about dyskinetic crises, their nature, and the appropriate actions to take during such episodes. By fostering an in-depth understanding of the distinctive characteristics of dyskinetic crises, caregivers will be able to recognize and respond swiftly to dyskinetic events. This proactive approach is invaluable, as it not only mitigates the immediate impact of dyskinetic crises but also potentially reduces the short- and long-term impacts. Early recognition leads to timely interventions, allowing for the administration of appropriate treatments, which may reduce the duration and severity of these episodes.

It is essential to acknowledge the limitations of our study. The difficulties in distinguishing dyskinetic crises from other neurological events, specifically epileptic seizures, require additional study. In addition, the lack of data on certain triggers, such as acute or chronic lack of sleep (which clonidine can ameliorate), necessitates further research to improve our understanding of the varied triggers that precipitate dyskinetic crises. Poor sleep hygiene and quality may precede a dyskinetic crisis by days or weeks, a phenomenon that can be monitored with easy-to-collate 24-h sleep–wake charts. Fragmented sleep patterns could represent early-warning ‘weather patterns’ of an impending dyskinetic crisis for which prophylactic nocturnal clonidine as a sleep regulator in *GNAO1*-RD requires further investigation.

Our findings challenge previously held beliefs regarding the correlation between *GNAO1*-RD severity and the incidence of dyskinetic crises. Milder phenotypes, which are frequently characterized by dystonic symptoms, do not always correlate with the presence of dyskinetic crises, emphasizing the need for additional research. Comprehensive long-term follow-up and natural history studies are required to identify specific risk factors, such as genotypes, age, and comorbid neurological characteristics, that may influence the occurrence and nature of dyskinetic crises.

In conclusion, our study represents an important step toward elucidating the complex nature of dyskinetic crises in *GNAO1*-RD. We have developed a comprehensive framework that not only defines dyskinetic crises but also outlines their clinical characteristics, triggers, and management. This foundational work lays the groundwork for improved clinical management, enhanced stakeholder communication, and future research endeavors.

## Data availability statement

The original contributions presented in the study are included in the article/Supplementary material, further inquiries can be directed to the corresponding author.

## Ethics statement

Ethical approval was not required for the study involving humans in accordance with the local legislation and institutional requirements. Written informed consent to participate in this study was not required from the participants or the participants’ legal guardians/next of kin in accordance with the national legislation and the institutional requirements. Written informed consent was obtained from the individual(s), and minor(s)’ legal guardian/next of kin, for the publication of any potentially identifiable images or data included in this article.

## Author contributions

JD: Writing – review & editing. CaR: Writing – review & editing, Project administration. DE-F: Writing – review & editing. ND: Writing – review & editing. SG: Writing – review & editing. GG: Writing – review & editing. MM: Writing – review & editing. ClR: Writing – review & editing. ES: Writing – review & editing. LP: Writing – review & editing. AK: Writing – review & editing. VL: Writing – review & editing. AR: Writing – review & editing. J-PL: Writing – review & editing. DD: Writing – review & editing. LC: Writing – review & editing. JO-E: Conceptualization, Data curation, Formal analysis, Methodology, Writing – original draft, Writing – review & editing.

## Supplementary material

SUPPLEMENTARY FIGURE S1 PRISMA flowchart.

SUPPLEMENTARY FIGURE S2 *GNAO1* variants in 102 reported cases of patients with dyskinetic crises.

SUPPLEMENTARY FIGURE S3 Pharmacological and surgical interventions reported by frequency in the literature. It is noteworthy that for most drugs, there is limited evidence, with only one or two articles describing the use of a specific medication in the context of dyskinetic crises.

SUPPLEMENTARY VIDEO S1 Shows multiple dyskinetic crises in a 12-year-old boy with the *de novo GNAO1* variant c.139A>G/p.S47G. These crises manifest as generalized dystonia and choreoathetosis lasting 5-30 minutes. The patient underwent elective GPi-DBS implantation at the age of 10 years, with a dramatic reduction in the frequency and severity of dyskinetic crises, from multiple and severe episodes per day to infrequent and more easily manageable episodes (<1 per month). The first video fragment shows a severe dyskinetic crisis at the age of 10 years immediately after GPi-DBS implantation, which evolved into dyskinetic status and was treated with sedation. The other three fragments show three dyskinetic crises that occurred between the ages of 11 and 12, triggered by fever. In addition to DBS, he is currently receiving treatment with tetrabenazine, lorazepam, and carbamazepine for epilepsy. His current *GNAO1*-RD severity score is 7 points (moderate). Part A. Background movement disorders with generalized dystonic postures.

SUPPLEMENTARY VIDEO S2 Shows a 19-year-old male diagnosed with the *de novo GNAO1* variant c.625C>T/p.R209C, exhibiting a dyskinetic crisis characterized by dyskinetic and choreoathetotic movements lasting from seconds to hours (Part B). Notably, this patient experienced infrequent and brief dyskinetic crises until the age of 18, which suddenly escalated in frequency, leading to daily occurrences. This escalation resulted in the loss of speech, motor autonomy, and the ability to walk. Dyskinesia was triggered by purposeful movements. The patient is currently undergoing treatment involving tetrabenazine, amitriptyline, bromazepam, and clonazepam during severe episodes. Additionally, he underwent GPi-DBS, resulting in a significant improvement in his condition. The *GNAO1*-RD severity score is 11 points (severe). Part A. Background movement disorders with dystonic postures in the upper limbs (hands).

SUPPLEMENTARY VIDEO S3 Shows a 10-year-old girl with the *de novo GNAO1* variant c.709G>A/p.Q237K exhibiting a dyskinetic crisis (Part B). This crisis manifests as generalized dystonia and choreoathetosis lasting 1.5 minutes. The patient experiences frequent daily crises triggered by emotions or fever. Autonomic symptoms, such as sweating and tachycardia, accompany these episodes. She is currently receiving treatment with clonidine, chloral hydrate, and clonazepam. Following a dystonic status, a GPi-DBS was implanted. Her current GNAO1 severity score is 9 points (severe). The data from this patient have been published as Patient 16 (P16) in the article by Dominguez-Carral et al. (2). Part A. Background movement disorders with oral dyskinesias.

SUPPLEMENTARY VIDEO S4 Presents a dyskinetic crisis (Part B) exhibited by a 7-year-old boy with the *de novo GNAO1* variant c.736G>A/p.Q246K. This crisis is characterized by generalized choreoathetosis that lasts between 30 seconds and 3 minutes. The patient experiences daily crises, mostly triggered by emotions. The autonomic symptoms of prolonged dyskinetic crises include profuse perspiration and tachycardia. Following a dyskinetic crisis, he often exhibits signs of fatigue. A dosage increase of scopolamine for chronic drooling resulted in a worsening of the frequency and severity of dyskinetic crises. He is currently on tetrabenazine and carbamazepine (for epileptic seizures). His current *GNAO1* severity score is 7.25 points (moderate). Part A. Background movement disorders with generalized dystonia.

SUPPLEMENTARY VIDEO S5 Shows a patient with a *de novo GNAO1* variant c.625C>T/p.R209C at age 23 years. Her baseline movement disorders are documented under Gpi-DBS. While sitting, the movement disorder has a generalized distribution, including cranial involvement (tongue protrusion dystonia) and dystonic postures of distal distribution for the four extremities. On the right side, prominent athetosis involving the two upper limbs is also demonstrated. While lying, dystonic postures with bilateral wrist flexion are visible, as are the right hallux erectus. The patient is capable of maintaining a lying position and performing a motor task (flexion or extension of the lower limb) without exacerbating the hyperkinetic features of her movement disorder. The second sequence shows the same patient at age 27 exhibiting a dyskinetic crisis with emotional stress as a potential triggering factor. The crisis manifests as generalized dystonia and choreoathetosis, including in the cranial segment, with a significant increase in the amplitude, intensity, and severity of the baseline movement disorder. Autonomic features with diaphoresis accompany the current crisis. Lying position is difficult to maintain because of the permanent and increased-amplitude dyskinetic phenomenology, prominent proximally at the four limbs, and associated cervical and trunk dystonia. The patient is currently receiving treatment with clonazepam, levetiracetam, and baclofen. She received Gpi-DBS in November 2007, at age 14 years, for ongoing status dyskineticus. Her current *GNAO1*-RD severity score is 8 (severe). The data from this patient have been published as Patient 3 (P3) in the article by Koy et al. (26).
