# Supplementary figures and images for: Dyskinetic crisis in GNAO1-related disorders: clinical perspectives and management strategies
Source: Front Neurol. 2024 Jun 6;15:1403815. doi: 10.3389/fneur.2024.1403815 (PMC11188927; doi:10.3389/fneur.2024.1403815)

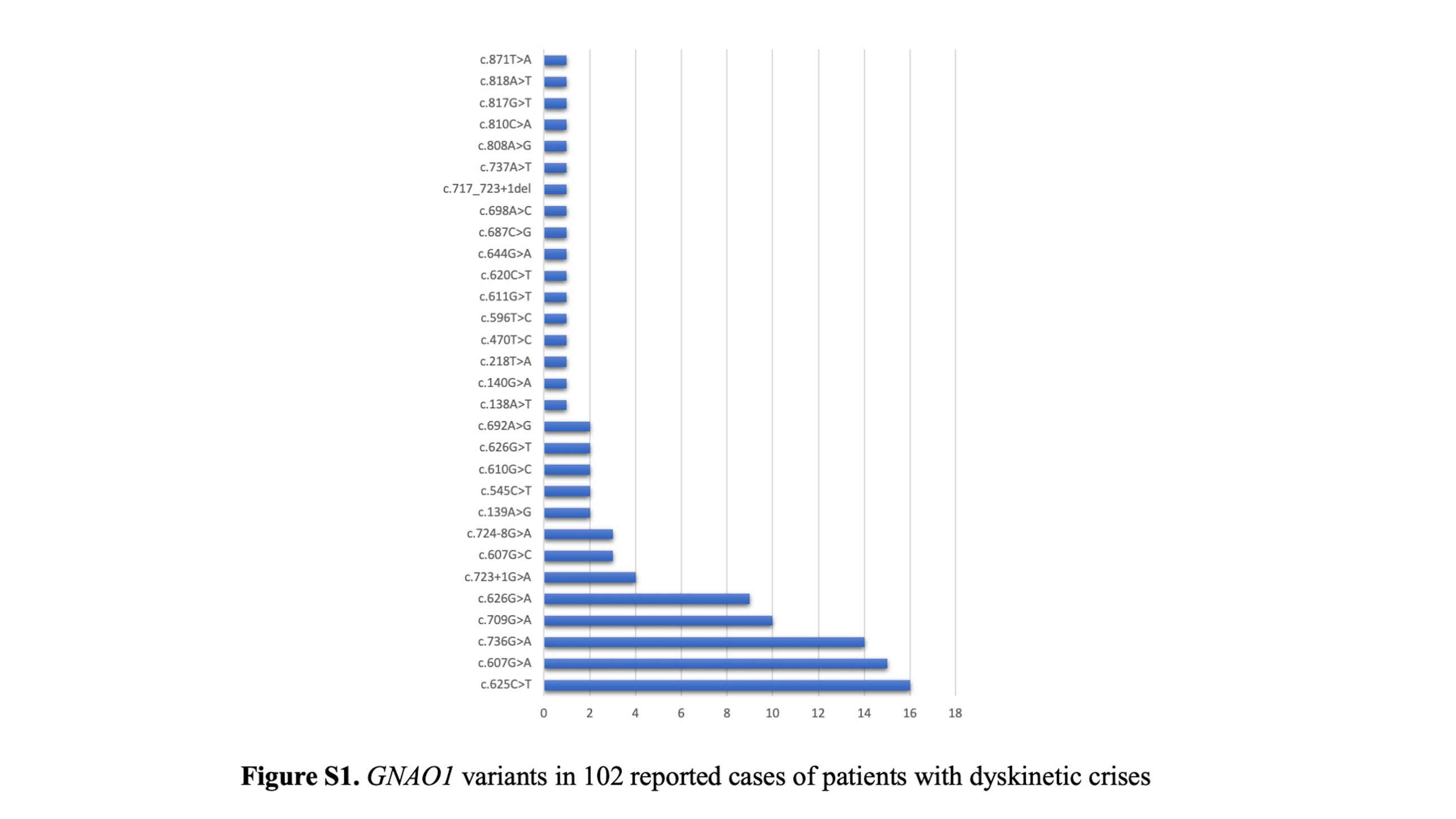

Supplement: SUPPLEMENTARY FIGURE S1 — PRISMA flowchart. [file Image_1.jpeg]

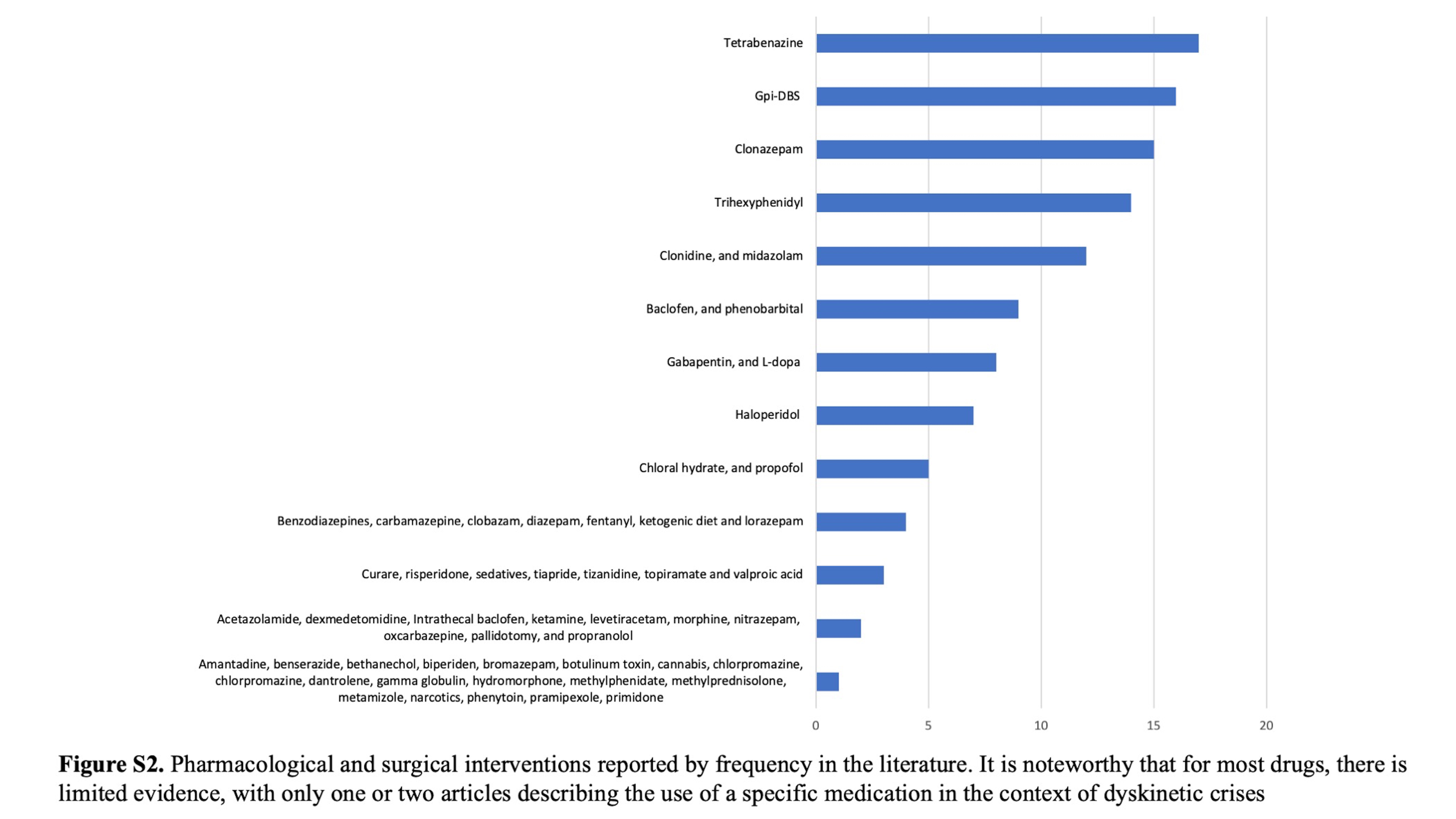

Supplement: SUPPLEMENTARY FIGURE S3 — Pharmacological and surgical interventions reported by frequency in the literature. It is noteworthy that for most drugs, there is limited evidence, with only one or two articles describing the use of a specific medication in the context of dyskinetic crises. [file Image_3.jpeg]
