# Supplementary material for: Dyskinetic crisis in GNAO1-related disorders: clinical perspectives and management strategies
Source: Front Neurol. 2024 Jun 6;15:1403815. doi: 10.3389/fneur.2024.1403815 (PMC11188927; doi:10.3389/fneur.2024.1403815)
Supplement: Supplementary file 9 [file Table_1.docx]

| **Table S1. *GNAO1* dyskinetic crisis Delphi consensus participants and their role** | |
| --- | --- |
| **Participant (n)** | **Role/other details** |
| Chair (1) | Responsible for agreeing the design of the Delphi consensus process, including selection of Delphi consensus panel members and the agreement threshold for statements questionnaire. Contributed to the development of the proto-statement questionnaire, initial statements, and revisions to statements that did not meet consensus Did not participate in the voting stage of the Delphi process |
| Steering committee members (N=2) | Responsible for agreeing the design of the Delphi consensus process, including selection of Delphi consensus panel members, questionnaire and statement development, and the agreement threshold for statements. Did not participated in the voting stage of the Delphi process |
| Panel members (N=13) | Participated in the voting stage of the Delphi process.  All Delphi panel members were identified based on their clinical expertise in the area of *GNAO1*-related disorders and selected based on their willingness to participate and with an aim to create good representation for geographic location and gender |

| **Table S2.** *GNAO1-RD* dyskinetic crisis statements on definition | First round | Second round |
| --- | --- | --- |
| Consensus statement | Level of agreement, i.e. n/N panel members who agreed | Level of agreement ,i.e., n/N panel members who agreed |
| 1. A dyskinetic crisis in *GNAO1*-RD is characterized by sudden, paroxysmal episodes of abnormal involuntary movements. | 9/13 (70%) | - |
| 1. Dyskinetic crisis episodes in *GNAO1*-RD typically involve multiple body regions, affecting both the upper and lower limbs, trunk, and face | 9/13 (70%) | - |
| 1. Dyskinetic crisis in *GNAO1*-RD is typically associated with dystonia, choreoathetosis, ballismus, or a combination of these movement disorders. | 11/13 (84%) | - |
| 1. Dyskinetic crisis episodes in *GNAO1*-RD often last for minutes to hours and can occur spontaneously or be triggered by various factors. | 9/13 (70%) | - |
| 1. During a dyskinetic crisis in *GNAO1*-RD, individuals may experience significant functional impairment and a loss of voluntary control over their movements. | 9/13 (70%) | - |
| 1. Dyskinetic crisis may occur multiple times per day or infrequently. | 7/13 (53%) | 6/8 (75%) |
| 1. In addition to the concept of dyskinetic crisis, in patients with *GNAO1*-RD, the term "dyskinetic status" should be introduced instead of "dystonic status | 7/13 (53%) | 6/8 (75%) |
| 1. Dyskinetic crisis in *GNAO1*-RD may exhibit a fluctuating course, with episodes varying in intensity, duration, and frequency over time. | 8/13 (61%) | 5/8 (63%) |
| 1. The term "dyskinetic status" should be used instead of "dystonic status" when there is a predominant component of chorea, athetosis, or ballismus rather than dystonia. | 8/13 (61%) | 5/8 (63%) |
| 1. During a dyskinetic crisis, individuals with *GNAO1*-RD should not exhibit impaired consciousness or impaired awareness. | 4/13 (30%) | 2/8 (25%) |
| 1. A dyskinetic crisis is distinguished by abrupt, paroxysmal occurrences of atypical involuntary movements that are distinct from the individual's typical basal motor patterns. | 7/13 (53%) | - |
| 1. Isolated orolingual dyskinesias are also a presentation of dyskinetic crises. | 7/13 (53%) | - |

| **Table S3.** *GNAO1-RD*  dyskinetic crisis statements on clinical features aside from MD | First round |
| --- | --- |
| Consensus statement | Level of agreement, i.e. n/N (5) panel members who agreed |
| 1. Dyskinetic crisis in *GNAO1*-RD may be accompanied by autonomic symptoms such as diaphoresis, tachycardia, or changes in blood pressure. | 10/13 (76%) |
| 1. Individuals experiencing a dyskinetic crisis in *GNAO1*-RD may have difficulties with speech and swallowing. | 11/13 (84%) |
| 1. Individuals with *GNAO1*-RD may experience fatigue or weakness after a dyskinetic crisis. | 11/13 (84%) |

| **Table S4.** *GNAO1-RD* dyskinetic crisis statements on triggers or precipitant factors | First round | Second round |
| --- | --- | --- |
| Consensus statement | Level of agreement, i.e., n/N panel members who agreed | Level of agreement, i.e., n/N panel members who agreed |
| 1. Emotional stress, such as anxiety or excitement, can frequently trigger dyskinetic crisis in individuals with *GNAO1*-related disorders. | 9/13 (70%) | - |
| 1. Infections, such as respiratory or urinary tract infections, can often precede or trigger dyskinetic crisis in individuals with *GNAO1*-RD. | 11/13 (84%) | - |
| 1. The treatment plan should include the avoidance of recognizable triggers for dyskinetic crises. | 11/13 (84%) | - |
| 1. Environmental factors, such as changes in temperature or humidity, can be triggers for dyskinetic crisis episodes in individuals with *GNAO1*-RD. | 6/13 (46%) | 5/8 (63%) |
| 1. Sleep disturbances or sleep deprivation are commonly reported triggers for dyskinetic crisis in individuals with *GNAO1*-RD. | 7/13 (53%) | 3/8 (38%) |
| 1. Certain medications or drug interactions may act as triggers for dyskinetic crisis episodes in individuals with *GNAO1*-RD. | 6/13 (46%) | 4/8 (50%) |
| 1. Physical exertion or strenuous activities, including exercise or prolonged periods of movement, may precipitate dyskinetic crisis in individuals with *GNAO1*-RD. | 4/13 (30%) | - |
| 1. Dietary factors, such as specific foods or food additives, have been reported to trigger dyskinetic crisis in some individuals with *GNAO1*-RD. | 2/13 (15%) | - |
| 1. Overstimulation or sensory overload, such as exposure to loud noises or bright lights, can provoke dyskinetic crisis in individuals with GNAO1-related disorders. | 2/13 (15%) | - |
| 1. Hormonal changes, such as those occurring during puberty or menstruation, may be associated with an increased frequency of dyskinetic crisis in individuals with *GNAO1*-RD. | 4/13 (20%) | - |
| 1. The different triggers for dyskinetic crisis can vary depending on the underlying movement disorder and individual factors. | 6/13 (46%) | - |

| **Table S5.** *GNAO1* dyskinetic crisis: differences compared to background MD | First round | Second round |
| --- | --- | --- |
| Consensus statement | Level of agreement, i.e., n/N panel members who agreed | Level of agreement, i.e., n/N panel members who agreed |
| 1. Dyskinetic crisis in *GNAO1*-RD often presents with paroxysmal dyskinesia-like movements, characterized by sudden, jerky, and non-rhythmic movements, that may differ from the slower, flowing movements seen in the basal state of the patient. | 4/13 (20%) | 4/8 (50%) |
| 1. Dyskinetic crisis in *GNAO1*-RD can be associated with autonomic symptoms, such as tachycardia, diaphoresis, or respiratory disturbances, which may not be commonly observed in other types of movement disturbances. | 6/13 (46%) | 5/8 (63%) |
| 1. Dyskinetic crisis in *GNAO1*-RD may display a more generalized distribution of abnormal movements, involving multiple body regions simultaneously, whereas other movement disturbances may exhibit a more focal or segmental distribution. | 7/13 (53%) | 2/8 (25%) |

| **Table S6.** *GNAO1-*RD dyskinetic crisis statements on distinctive patterns or variations | First round | Second round |
| --- | --- | --- |
| Consensus statements | Level of agreement, i.e., n/N panel members who agreed | Level of agreement, i.e., n/N panel members who agreed |
| 1. Certain disease severity levels within *GNAO1*-RD may display a more severe or refractory form of dyskinetic crisis, characterized by longer duration, higher frequency, and a limited response to conventional treatments. | 7/13 (53%) |  |
| 1. Dyskinetic crisis in *GNAO1*-RD may exhibit variations in severity, frequency, and specific motor manifestations across different age groups, ranging from infancy to adulthood. | 4/13 (20%) |  |
| 1. Dyskinetic crises decrease with age | 1/13 (8%) |  |
| 1. Some individuals with *GNAO1*-RD may exhibit a distinct age-related evolution of dyskinetic crisis, with a transition from predominantly choreiform or athetoid movement presentations in infancy to dystonic presentations in later childhood or adolescence. | 3/13 (23%) |  |
| 1. There is a genotype-phenotype correlation, with more frequent and severe dyskinetic crises associated with certain variants in GNAO1. | 3/13 (23%) |  |
| 1. Adolescents and adults with *GNAO1*-RD with a more dystonic phenotype may not experience dyskinetic crisis. | 5/13 (38%) |  |
| 1. The presence of accompanying neurological features, such as cognitive impairment, epilepsy, or developmental delay, may influence the clinical presentation and course of dyskinetic crisis in *GNAO1*-RD. | 3/13 (23%) |  |
| 1. Infants with *GNAO1*-RD often present with dyskinetic crisis episodes characterized by generalized dystonia, chorea, or ballismus, accompanied by autonomic features such as respiratory distress or feeding difficulties. | 4/13 (20%) | 2/8 (25%) |
| 1. Certain disease severity levels within *GNAO1*-RD may display a more severe or refractory form of dyskinetic crisis, characterized by longer duration, higher frequency, and a limited response to conventional treatments. | - | 4/8 (50%) |

| **Table S7.** *GNAO1* dyskinetic crisis statements on diagnostic criteria and guidelines | First round | Second round |
| --- | --- | --- |
| Consensus statement | Level of agreement, i.e., n/N panel members who agreed | Level of agreement, i.e., n/N panel members who agreed |
| 1. Dyskinetic crisis should be considered in individuals with *GNAO1*-RD who present with acute, paroxysmal, and involuntary dyskinetic movements. | 9/13 (70%) | - |
| 1. There is currently no specific diagnostic criteria or guidelines exclusively tailored to dyskinetic crisis in *GNAO1*-RD | 11/13 (84%) | - |
| 1. Video documentation of dyskinetic crisis episodes can aid in the diagnostic process, providing visual evidence of the nature, frequency, and characteristics of the abnormal movements. | 11/13 (84%) | - |
| 1. Longitudinal observation and monitoring of dyskinetic crisis episodes, along with comprehensive clinical assessments, can contribute to establishing diagnostic patterns. | 11/13 (84%) | - |
| 1. During dyskinetic crisis, awareness is typically preserved and movements affect both hemispheres in an inconsistent pattern, whereas epileptic seizures are highly stereotyped and, when bilateral, awareness will be impaired. | 9/13 (70%) | - |
| 1. A differential diagnosis of dyskinetic crisis should involve ruling out other potential causes of paroxysmal movement disturbances, such as epileptic seizures. | 9/13 (70%) | - |
| 1. Electrophysiological studies, such as electroencephalography (EEG), may be useful to differentiate dyskinetic crisis from epileptic seizures, which typically exhibit characteristic EEG abnormalities. | 4/13 (20%) | 3/8 (38%) |

| **Table S8.** *GNAO1-RD* dyskinetic crisis statements on potential short- and long-term complications | First round | Second round |
| --- | --- | --- |
| Consensus statement | Level of agreement, i.e., n/N panel members who agreed | Level of agreement, i.e., n/N panel members who agreed |
| 1. Dyskinetic crisis can interfere with activities of daily living and functional independence, resulting in a decreased quality of life for individuals with *GNAO1*-RD. | 12/13 (92%) | - |
| 1. Dyskinetic crisis may be associated with an increased risk of joint dislocations. | 5/13 (38%) | - |
| 1. Dyskinetic crisis in *GNAO1*-RD may lead to a progressive deterioration in motor function and result in significant motor disability over time. | 7/13 (53%) | 3/8 (38%) |

| **Table S9.** *GNAO1* dyskinetic crisis statements on management strategies and interventions | First round | Second round |
| --- | --- | --- |
| Consensus statement | Level of agreement, i.e., n/N panel members who agreed | Level of agreement, i.e., n/N panel members who agreed |
| 1. Patients with dyskinetic crises should receive the same care (indication of hospitalization, medication, laboratory, etc.) as described in the Dystonia Action Plan. | 10/13 (76%) | - |
| 1. Rhabdomyolysis, electrolyte, and renal abnormalities should be ruled out in patients with prolonged dyskinetic crises. | 11/13 (84%) | - |
| 1. Deep brain stimulation (DBS) has shown promise as a therapeutic option for individuals with severe and refractory dyskinetic crisis in *GNAO1*-RD, providing significant improvement in motor symptoms. | 10/13 (76%) | - |
| 1. In patients with frequent dyskinetic crises or those requiring hospitalization, deep brain stimulation (DBS) should be considered early in the treatment plan. | 9/13 (70%) | - |
| 1. Regular monitoring and adjustment of medication regimens, guided by clinical response and side effect profiles, are important in optimizing the management of dyskinetic crisis in *GNAO1*-RD. | 11/13 (84%) | - |
| 1. Parent and caregiver education and support are vital components of managing dyskinetic crisis, ensuring a coordinated approach, and promoting adherence to treatment plans. | 11/13 (84%) | - |
| 1. Individualized treatment plans, tailored to the specific needs of each patient, combining different therapeutic modalities, including medications, therapies, and assistive devices, have been effective in managing dyskinetic crisis in *GNAO1*-RD. | 7/13 (53%) | 7/8 (88%) |
| 1. Dyskinetic crisis in *GNAO1*-RD often do not respond well to conventional antiepileptic medications used for epileptic seizures associated with this condition. | 6/13 (46%) | 4/8 (50%) |
| 1. The patient's action plan should include the use of oral or rectal benzodiazepines, similar to the management of a prolonged epileptic seizure. | 6/13 (46%) | - |
| 1. Dyskinetic crisis in *GNAO1*-RD typically subside spontaneously. | 0/13 (13%) | - |
| 1. Non-pharmacological approaches, such as behavioral interventions, may help in mitigating dyskinetic crisis episodes and improving overall functioning in individuals with *GNAO1*-RD. | 4/13 (20%) | - |
| 1. In postoperative patients with deep brain stimulation (DBS), there is often a change in the semiology of dyskinetic crises, with improved involvement of the limbs and a greater predominance in the face and orolingual area | 5/13 (38%) | - |
| 1. Assistive devices, such as orthoses or adaptive equipment, can aid in enhancing mobility, reducing the impact of involuntary movements, and improving the overall quality of life for individuals experiencing dyskinetic crisis. | 4/13 (20%) | - |
| 1. Pharmacological interventions, such as benzodiazepines or other GABAergic agents, have demonstrated efficacy in reducing the frequency and severity of dyskinetic crisis episodes in *GNAO1*-RD. | 7/13 (53%) | 1/8 (13%) |

| **Table S10.** *GNAO1*-RD dyskinetic crisis: statements on additional factors and considerations | First round | Second round |
| --- | --- | --- |
| Consensus statement | Level of agreement, i.e., n/N panel members who agreed | Level of agreement, i.e., n/N panel members who agreed |
| 1. At the time of GNAO1-RD diagnosis, parental or caregiver education should begin, including information about dyskinetic crises, what they are, and what should be done when the child experiences a crisis. | 11/13 (84%) | - |
| 1. The duration and frequency of dyskinetic crisis episodes should be considered when characterizing the condition, as they can vary among individuals with GNAO1-RD. | 8/13 (61%) | 6/8 (75%) |
| 1. The age of onset and disease progression may influence the manifestation and severity of dyskinetic crisis in individuals with GNAO1-RD. | 6/13 (46%) | 2/8 (25%) |
| 1. The presence of comorbidities, such as epilepsy or intellectual disability, may contribute to the complexity and clinical presentation of dyskinetic crisis. | 6/13 (46%) | 2/8 (25%) |
| 1. Parents or primary caregivers are often able to differentiate a dyskinetic crisis from an epileptic seizure. | 7/13 (53%) | 4/8 (50%) |

| **Table S11.** *GNAO1*-RD dyskinetic crisis: modified statements for the second round | Second round |
| --- | --- |
| Consensus statement | Level of agreement, i.e., n/N panel members who agreed |
| 1. A dyskinetic crisis is characterized by sudden, episodic episodes of atypical involuntary movements that significantly deviate from the usual baseline motor patterns of the individual. | 5/8 (62%) |
| 1. The occurrence of dyskinetic crises generally decreases as patients advance in age. | 1/8 (13%) |
| 1. In certain cases of GNAO1-RD, there may be an observable age-related progression of dyskinetic crises. This progression can involve a shift from primarily choreiform or athetoid movements during infancy to dystonic presentations in later childhood or adolescence. | 4/8 (50%) |
| 1. Individuals with GNAO1-related disorders with a more dystonic phenotype or hypokinetic features may not experience a dyskinetic crisis. | 2/8 (25%) |
| 1. Dyskinetic crises in individuals with GNAO1-related disorders usually resolve spontaneously or with the suppression of the triggering factor. | 1/8 (13%) |
| 1. In postoperative patients with deep brain stimulation (DBS), there is often a change in the semiology of dyskinetic crises, with improved involvement of the limbs. | 3/8 (38%) |
| 1. In postoperative patients with deep brain stimulation (DBS), there can be an increase in facial and orolingual dyskinesia. | 1/8 (13%) |

| **Table S12.** *GNAO1*-RD dyskinetic crisis: new statements for the second round | Second round |
| --- | --- |
| Consensus statement | Level of agreement, i.e., n/N panel members who agreed |
| 1. The definition of a dyskinetic crisis should include a specific time-frame. | 3/8 (38%) |
| 1. The definition of a dyskinetic crisis should be based on the type of movements observed, irrespective of the duration of the episode. | 1/8 (13%) |
| 1. The definition of a dyskinetic crisis should take into account its frequency. | 1/8 (13%) |
| 1. The definition of a dyskinetic crisis should not be contingent upon frequency; even if a patient experiences daily dyskinetic crises, they are still considered dyskinetic crises. | 2/8 (25%) |
| 1. The terminology 'dyskinetic spells' should be incorporated, defined as brief, paroxysmal episodes of abnormal involuntary movements, typically lasting seconds to minutes, which are distinct from the more sustained and intense dyskinetic crisis characterized by prolonged, often hours-long, atypical movements. | 4/8 (50%) |
| 1. Dyskinetic crises may also manifest as focal events. | 3/8 (38%) |
| 1. The term 'status dystonicus' encompasses 'dyskinetic status,' and therefore, there should be no differentiation, and the term 'dyskinetic status' should not be used. | 0/8 (0%) |
| 1. Hyperthermia should be included as a defining factor in the characterization of dyskinetic crisis, as it may be a significant clinical feature: e.g., Dyskinetic crisis in GNAO1-RD may be accompanied by autonomic symptoms such as diaphoresis, tachycardia, hyperthermia, or changes in blood pressure. | 5/8 (63%) |
| 1. Pain can trigger dyskinetic crisis in individuals with *GNAO1*-RD. | 6/8 (75%) |
| 1. The different triggers for dyskinetic crisis can vary depending on individual factors. | 5/8 (63%) |
| 1. Most frequently generalized, intense, faster movements than baseline movements" should be incorporated into the differential diagnosis of dyskinetic crisis. | 2/8 (25%) |
| 1. Identifying all potential triggers for dyskinetic crises in *GNAO1*-RD may pose a challenge, as experience is often limited and primarily based on anecdotal evidence. | 4/8 (50%) |
| 1. Autonomic symptoms, in isolation, along with increased movements, do not by themselves define a dyskinetic crisis. Autonomic symptoms should be recognized as potential features of various movement disorders, not exclusive to dyskinetic crisis, warranting a comprehensive evaluation. | 6/8 (75%) |
| 1. Dyskinetic crisis may disrupt the ability to maintain oral feeding and should be considered as a factor affecting nutritional management. | 6/8 (75%) |
| 1. Dyskinetic crisis may be associated with an increased risk of falls and/or injuries (pain, tongue biting, joint dislocations). | 7/8 (88%) |

| **Table S13.** *GNAO1*-RD dyskinetic crisis: new statements for the second round | Second round |
| --- | --- |
| Consensus statement | Level of agreement, i.e., n/N panel members who agreed |
| 1. The term 'dyskinetic crisis' should remain unchanged as originally suggested | 7/10 (70%) |
