# Supplementary material for: Dyskinetic crisis in GNAO1-related disorders: clinical perspectives and management strategies
Source: Front Neurol. 2024 Jun 6;15:1403815. doi: 10.3389/fneur.2024.1403815 (PMC11188927; doi:10.3389/fneur.2024.1403815)
Supplement: Supplementary file 10 [file Table_2.docx]

| **Supplemental Data.** Results of the literature review. | | | | | | |
| --- | --- | --- | --- | --- | --- | --- |
| **Reference** | **Name of the phenomenon** | **Genotype** | **MD Phenomenology** | **Triggers** | **Management** | **Other comments** |
| Akasaka, 2020 ^1^ | “continuous, generalized  involuntary movements” | c.626G>A (1) | Severe mixed involuntary movement of all four extremities and face | Viral infection, influenza type B infection | MDZ, dantrolene, triclofos sodium, chloral hydrate, plasma Exchange (for rhabdomyolysis) | Hyperthermia 41ºC, rhabdomyolysis (256,725 UI/L),  nasogastric tube |
| Ananth, 2016 ^2^ | Exacerbations or spells | c.625C>G (1)  c.626G>A (1)  c.736G>A (4) | Chorea involving all four extremities and face, and ballismus | Excitement, emotional, stress, viral illness, urinary tract infection, Clostridium difficile infection, bowel movements | Clonazepam, clonidine, trazodone, MDZ, **risperidone**, tetrabenazine, valproic acid, bethanechol, lorazepam, trihexyphenidyl, dexmedetomidine, propofol, clobazam, levetiracetam, topiramate, **baclofen,** haloperidol, diazepam, pentobarbital, fentanyl. Vecuronium, opiods, oxcarbazepine | Tachycardia,  hypertension, hyperthermia, diaphoresis,  dehydration,  rhabdomyolysis,  pressure ulcers,  femur fractures  Death (respiratory complication, sepsis) |
| Arisaka 2021 ^3^ | NA | c.607G>A (1) | Irregular, violent,  choreo-ballistic movements, including oromandibular movements,  while awake | NA | High-dose phenobarbital, gabapentin, clonazepam, pallidal electrocoagulation (partially effective) | Hyperthermia, injury |
| Benato, 2019 ^4^ | Dystonic–dyskinetic movements, hyperkinesia | c.736G>A (1) | Dystonia, chorea and ballismus involving the limbs | Respiratory illness | MDZ, propofol, thiopental, neuromuscular blockade, GPi-DBS | Gastric feeding tube, hyperthermia  rhabdomyolysis |
| Chaib 2022 ^5^ | Dystonic  storm | c.626G>T (1) | Episodes of varying intensity of dystonic movements  of her arms and legs and later involving also the trunk. | After a tooth extraction under general anesthesia | Clonidine, midazolam, morphine, hydromorphone, clobazam, tetrabenazine, gabapentin, GPi-DBS | Rhabdomyolysis (>100.000 UI/L), hyperkalemia (6.9 mmol/l), renal impairment,  acute colitis, pneumatosis hepatis |
| Danhofer 2021 ^6^ | Worsening of extrapyramidal symptomatology | c.625C>T (1) | Severe biballistic symptomatology (DSAP 3): almost  continuous generalized ballistic movements combined with dystonic  postures, which were very painful and limited his normal  activity, feeding, or sleep | Respiratory  infection | Clonazepam, midazolam, baclofen, phenobarbital, propofol, tetrabenazine, tiapride, valproic acid, gabapentin, GPi-DBS | NA |
| Danti, 2017^7^ | Severe episodes  of paroxysmal choreoathetosis | c.139A>G (1)  c.625C>T (1)  c.723+1G>A (1)  c.737A>G (1) | Attacks frequently  presented in clusters, lasting minutes,  hours or weeks, or months | Emotion, infection, high temperature, intention, and purposeful  movements | Sedation, GPi-DBS | Tracheostomy, gastrostomy, rhabdomyolysis, autonomic instability, sweating,  dehydration  Death (multisystemic failure) |
| Dominguez 2023 ^8^ | Dyskinetic crisis | c.545C>T (2)  c.596T>C (1)  c.626G>A (1)  c.692A>G (1)  c.871T>A (1)  c.607G>C (2)  c.625C>T (1)  c.709G>A (1) | Dyskinetic crisis: episodes of generalized choreodystonia | Bathing, emotions, temperature changes, mobilizations, unexpected sounds, gastrointestinal stimuli, stress, stimulation, without a clear cause during sleep | Baclofen, chloral hydrate,  clonidine, tetrabenazine, carbamazepine, trihexyphenidyl, clonazepam, clobazam, tizanidine, L-dopa, Gpi-DBS | Dehydration, profuse perspiration, hypernatremia, hyperthermia |
| Dzinovic 2021 ^9^ | Exacerbations of hyperkinetic movements | c.625C>T (1) | Dystonia was accompanied  by ballism and choreatic movements | Fever, infection | NA | NA |
| Fung 2022 ^10^ | Worsening of  hyperkinetic movement | c.709G>A (1) | Near continuous choreoathetoid  movement involving the whole body | Enterovirus infection | Midazolam, fentanyl, propofol, clonazepam, trihexyphenidyl, baclofen, tetrabenazine, clonidine, chloral hydrate, Gpi-DBS | Rhabdomyolysis, pneumonia |
| Gambardella 2023 ^11^ | Dyskinetic status | c.139A>G (1)  c.607G>C (1) | NA | NA | Clonazepam, tetrabenazine, baclofen, nitrazepam | NA |
| Garofalo 2023 ^12^ | Episodic deterioration  of the movement disorder | c.626G>A (1) | Severe bilateral choreatic  movements of increasing frequency, disturbing sleep. A combination of continuous, severe  generalized chorea, dystonia, and ballism. | Heat (i.e., fever, hot weather) or infections | Gabapentin, clonazepam, trihexyphenidyl, tetrabenazine, haloperidol, propofol, fentanyl, clonidine, phenobarbital, Lorazepam | Creatine kinase was 302 U/L (normal range: 20–30 U/L). |
| Graziola 2021 ^13^ | Refractory status  dystonicus | c.709G>A (2) | NA | NA | Trihexyphenidyl, tetrabenazine, Gpi-DBS | NA |
| Honey 2018 ^14^ | Dystonic storm | c.626G>T (1) | Severe dyskinetic movements of all  four limbs and his mouth (chorea, ballismus, orofacial dyskinesia,  and dystonia). | Acute infection | MDZ, ketamine, clonidine, dexmedetomidine, lorazepam, Gpi-DBS | Rhabdomyolysis (16.000 UI/L), lactic acidemia 13.7 mmol/L (0.7-2.1), increased plasma ammonia 43 mmol/L  (9-33) |
| Kim 2020 ^15^ | Intermittent  hyperkinesia | c.626G>A (1) | Intermittent  hyperkinesia with brief jerking and truncal dystonia | Emotional upset | NA | NA |
| Krygier 2022 ^16^ | NA | c.607G>A (1) | Generalized  chorea, that was initially intermittent but have increased in frequency  and severity over time. | NA | Primidone, gabapentin | NA |
| Koy 2018 ^17^ | Hyperkinetic state,  hyperkinetic crises, recurrent episodes of hyperkinesia | c.723+1G>T(1)  c.610G>C(1)  c.625C>T(2)  c.709G>A(2) | Recurrent episodes of hyperkinesia with dystonia,  choreoathetosis, ballism, severe orofacial dyskinesia | Change of positioning, voluntary movements,  illnesses with or without fever, emotional agitation, drugs (metopimazine, trihexyphenidyl) | High doses of sedatives, benzodiazepines and muscle relaxants, tetrabenazine, Gpi-DBS | Autonomic features (hyperthermia, hypertension, sweating, tachycardia), rhabdomyolysis, dehydration |
| Kwong 2021 ^18^ | Status dystonicus | c.625C>T (1) | NA | NA | Tetrabenazine | Sudden death |
| Lee 2021 ^19^ | Intractable dystonia | c.607G>A(1) | NA | NA | NA | NA |
| Li 2023 ^20^ | Severe chorea and dystonia | c.140G>A(1)  c.607G>A(1)  c.717_723+1del (1)  c.709G>A (1)  c.808A>G (1) | Severe chorea and dystonia | Fever, emotional stimulus | L-dopa, gabapentin, clonazepam, risperidone, tetrabenazine, ketogenic diet, tizanidine, trihexyphenidyl | Death (2) |
| Ling 2023 ^21^ | NA | c.709G>A (1) | Restlessness, involuntary movements of the limbs, body  writhing, profuse sweating, and unresponsiveness | NA | **Oxcarbazepine**, tiapride, methylprednisolone, gamma globulin, midazolam, phenobarbital | Sweating |
| Malaquias 2019 ^22^ | Fluctuations | c.625C>T (1) | Severe episodes of persistent, rapid,  irregular choreodystonic movements | Infections (typhoid fever) | L-dopa, clonazepam, trihexyphenidyl | Dysautonomic features  (palpitations, chest pain), anxiety, and oculogyric crises |
| Marecos 2018 ^23^ | Episodes | c.626G>A (1) | Dystonia, chorea and ballismus | Excitation, stress | Carbamazepine, clonidine, acetazolamide, risperidone, trihexyphenidyl, haloperidol, tetrabenazine, propranolol, valproate, chlorpromazine, MDZ, ketamine, chloral hydrate, biperiden, ketogenic diet, metamizole | Rhabdomyolysis (11.000 UI/L), prerenal insufficiency |
| Novelli 2023 ^24^ | MD fluctuations, periodic exacerbation of hyperkinetic  and involuntary movements dystonic–dyskinetic status | c.607G>A (2)  c.625C>T (1)  c.626G>A (1)  c.723+1G>A(1)  c.736G>A(3) | Dystonia,  chorea,  dyskinesia | NA | Nitrazepam, tetrabenazine, trihexyphenidyl, clonazepam, clonidine, benserazide, baclofen, clobazam, levetiracetam, L-dopa, carbamazepine, benzodiazepines, intratecal baclofen pump, Gpi-DBS | Sweating and flushing |
| Saitsu 2016 ^25^ | Status dystonicus | c.625C>T (1)  c.736G>A (1) | Chorea, dystonia | After orthopedic surgery, infection, fever | Sedative, | Fever/ Hyperthermia, vomiting, acidosis  Tracheostomy, enterostomy |
| Sakamoto 2017 ^26^ | Chorea episodes | c.625C>T (1) | Episodes occurred several times per day and usually lasted up to 30 min | Infection, pain, pyrexia, menstruation | MDZ, diazepam, midazolam, phenobarbital, haloperidol, tiapride, bromazepam, **topiramate** | Hyperthermia, rhabdomyolysis (neuroleptic malignant syndrome?) |
| Schirinzi 2019 ^27^ | Dyskinetic status, paroxysmal episodes, dyskinetic episodes, movement disorders emergency | c.625C>T (1)  c.607G>A (2)  c.818A>T (1) | Continuous generalized choreo-ballistic hyperkinesia (especially at level of face and lower limbs), which were responsible for the rapid loss of walking ability. Generalized dystonia with paroxysmal ballism and chorea with fluctuating course. Paroxysmal episodes of generalized dystonia, together with rapid dyskinetic movements of upper extremities appeared. These attacks, 3-10 minutes long, presented several times a day, during wakefulness, | Febrile illness, painful stimuli, action- or stimulus- induced. | Haloperidol, midazolam, propofol, phenytoin, baclofen, clonazepam, levodopa, tetrabenazine, clonidine, lorazepam, phenobarbital, trihexyphenidyl, curare, bilateral pallidotomy | Headache, irritability, obstinate constipation, rhabdomyolysis  Mechanical ventilation, dialysis |
| Schorling 2017 ^28^ | Episodes of dyskinetic movements | c.607G>A (2)  c.736G>A (2) | Pronounced  dyskinetic and atactic features including his tongue.  The dystonic involuntary movements mimicked  tonic–epileptic seizures but had no epileptic correlate in  repetitive video-EEG recordings. | NA | Gabapentin, trihexyphenidyl, baclofen, benzodiazepines, L-dopa | NA |
| Thiel 2023^29^ | Hyperkinetic crisis | c.138A>T(1)  c.218T>A (1)  c.607G>A (4)  c.610G>C (1)  c.625C>T (3)  c.626G>A (1)  c.709G>A (1)  c.723+1G>T (1)  c.736G>A (2) | Hyperkinetic crisis, paroxysmal chorea | Infection | Benzodiazepines, tizanidine, cannabis, baclofen, phenobarbital, tetrabenazine, trihexyphenidyl, gabapentin, topiramate, chloral hydrate, Gpi-DBS | NA |
| Waak 2020 ^30^ | Exacerbations | c.625C>T (1) c.709G>A (1)  c.736G>A (1) | Violent mixed hyperkinesis  (chorea, dystonia and orofaciolingual  dyskinesia). | Fever | Clonazepam, tetrabenazine, phenobarbitone, intrathecal baclofen, clonidine, Gpi-DBS | Joint dislocations and orolingual and superficial injuries,  hyperthermia,  rhabdomyolysis, dehydration,  prerenal failure.  Movements impaired  communication, motor function, sleep  and exacerbated hypotonia. |
| Wirth 2023 ^31^ | Acute exacerbation | c.644G>A(1)  c.724-8G>A(1)  c.737A>T (1) | NA | NA | L-dopa, trihexyphenidyl, methylphenidate, amantadine, haloperidol, tetrabenazine, clonidine, Gpi-DBS | NA |
| Yamamoto 2022 ^32^ | Refractory chorea episode | c.611G>T (1) | Severe, generalized chorea, each lasting  several weeks | NA | Diazepam, clonidine, narcotics, Gpi-DBS | Developmental regression, fractures, rhabdomyolysis, skin breakdown, weight loss, death. |
| Yamashita 2020 ^33^ | Exacerbation of dyskinesia | c.620C>T (1) | Choreoathetosis of the limbs and trunk, and dystonia. | NA | Tetrabenazine, trihexyphenidyl, clonazepam,  botulinum toxin, and pramipexol, Gpi-DBS | NA |
| Yang 2021 ^34^ | NA | c.470T>C(1)  c.607G>A(1)  c.687C>G(1)  c.692A>G(1)  c.724-8G>A (2)  c.810C>A(1)  c.817G>T(1) | Brief focal or truncal dystonia. Severe and persistent involuntary movements. | Sound  stimulus, emotional disturbance | Clonazepam, L-dopa, trihexyphenidyl, ketogenic diet | Died (1) |
| Yilmaz 2016^35^ | Movement disorders | c.698A>C (1) | Choreoathetosis with marked orofacial dyskinesia | Fever, infections | Clonazepam, haloperidol, carbamazepine, acetazolamide, diazepam, fentanyl, Gpi-DBS | Hyperthermia 42ºC,  rhabdomyolysis, tracheostomy |

References

1. Akasaka M, Kamei A, Tanifuji S, et al. GNAO1 mutation-related severe involuntary movements treated with gabapentin. Brain Dev. 2021;43(4):576–579.

2. Ananth AL, Robichaux-Viehoever A, Kim YM, et al. Clinical Course of Six Children with GNAO1 Mutations Causing a Severe and Distinctive Movement Disorder. Pediatr. Neurol. 2016;59:81–84.

3. Arisaka A, Nakashima M, Kumada S, et al. Association of early-onset epileptic encephalopathy with involuntary movements – Case series and literature review [Internet]. Epilepsy Behav. Reports 2021;15:100417.Available from: https://doi.org/10.1016/j.ebr.2020.100417

4. Benato A, Carecchio M, Burlina A, et al. Long-term effect of subthalamic and pallidal deep brain stimulation for status dystonicus in children with methylmalonic acidemia and GNAO1 mutation. J. Neural Transm. 2019;126(6):739–757.

5. Chaib H, Schoene-Bake JC, Saryyeva A, et al. DBS emergency surgery for treatment of dystonic storm associated with rhabdomyolysis and acute colitis in DYT-GNAO1. Child’s Nerv. Syst. 2022;38(9):1821–1824.

6. Danhofer P, Zech M, Bálintová Z, et al. Brittle Biballism-Dystonia in a Pediatric Patient with GNAO1 Mutation Managed Using Pallidal Deep Brain Stimulation. Mov. Disord. Clin. Pract. 2021;8(1):153–155.

7. Danti FR, Galosi S, Romani M, et al. GNAO1 encephalopathy: Broadening the phenotype and evaluating treatment and outcome. Neurol. Genet. 2017;3(2)

8. Domínguez-Carral J, Ludlam WG, Junyent Segarra M, et al. Severity of GNAO1-Related Disorder Correlates with Changes in G-Protein Function. Ann. Neurol. 2023;

9. Dzinovic I, Škorvánek M, Necpál J, et al. Dystonia as a prominent presenting feature in developmental and epileptic encephalopathies: A case series. Park. Relat. Disord. 2021;90(August):73–78.

10. Fung EL, Mo CY, Fung ST, Chan AY, Lau KY, Chan EK, Chan DY, Zhu XL, Chan DT PW. Deep brain stimulation in a young child with GNAO1 mutation - Feasible and helpful. Surg. Neurol. Int. 2022;13:285.

11. Gambardella ML, Pede E, Orazi L, et al. Visual Function in Children with GNAO1-Related Encephalopathy. Genes (Basel). 2023;14(3)

12. Garofalo M, Beudel M, Dijk JM, et al. Elective and Emergency Deep Brain Stimulation in Refractory Pediatric Monogenetic Movement Disorders Presenting with Dystonia: Current Practice Illustrated by Two Cases. Neuropediatrics 2023;54(1):44–52.

13. Graziola F, Garone G, Grasso M, Capuano A. Cognitive assessment in GNAO1 neurodevelopmental disorder using an eye tracking system. J. Clin. Med. 2021;10(16):1–8.

14. Honey CM, Malhotra AK, Tarailo-Graovac M, et al. GNAO1 Mutation–Induced Pediatric Dystonic Storm Rescue With Pallidal Deep Brain Stimulation. J. Child Neurol. 2018;33(6):413–416.

15. Kim SY, Shim YK, Ko YJ, et al. Spectrum of movement disorders in GNAO1 encephalopathy: in-depth phenotyping and case-by-case analysis. Orphanet J. Rare Dis. 2020;15(1):1–6.

16. Krygier M, Zawadzka M, Sawicka A, Mazurkiewicz-Bełdzińska M. Reflex seizures in rare monogenic epilepsies. Seizure 2022;97(January):32–34.

17. Koy A, Cirak S, Gonzalez V, et al. Deep brain stimulation is effective in pediatric patients with GNAO1 associated severe hyperkinesia. J. Neurol. Sci. 2018;391:31–39.

18. Kwong AKY, Tsang MHY, Fung JLF, et al. Exome sequencing in paediatric patients with movement disorders. Orphanet J. Rare Dis. 2021;16(1):1–12.

19. Lee J, Park JE, Lee C, et al. Genomic Analysis of Korean Patient With Microcephaly. Front. Genet. 2021;11(January)

20. Li Y, Chen H, Li L, et al. Phenotypes in children with GNAO1 encephalopathy in China. Front. Pediatr. 2023;11(August)

21. Ling W, Huang D, Yang F, et al. Treating GNAO1 mutation-related severe movement disorders with oxcarbazepine: a case report. Transl. Pediatr. 2022;11(9):1577–1587.

22. Malaquias MJ, Fineza I, Loureiro L, et al. GNAO1 mutation presenting as dyskinetic cerebral palsy. Neurol. Sci. 2019;40(10):2213–2216.

23. Marecos C, Duarte S, Alonso I, et al. GNAO1: Un nuevo gen a considerar en la distonía temprana de la infancia. Rev. Neurol. 2018;66(9):321–322.

24. Novelli M, Galosi S, Zorzi G, et al. GNAO1-related movement disorder: An update on phenomenology, clinical course, and response to treatments. Park. Relat. Disord. 2023;111(April):105405.

25. Saitsu H, Fukai R, Ben-Zeev B, et al. Phenotypic spectrum of GNAO1 variants: Epileptic encephalopathy to involuntary movements with severe developmental delay. Eur. J. Hum. Genet. 2016;24(1):129–134.

26. Sakamoto S, Monden Y, Fukai R, et al. A case of severe movement disorder with GNAO1 mutation responsive to topiramate. Brain Dev. 2017;39(5):439–443.

27. Schirinzi T, Garone G, Travaglini L, et al. Phenomenology and clinical course of movement disorder in GNAO1 variants: Results from an analytical review. Park. Relat. Disord. 2019;61(November 2018):19–25.

28. Schorling DC, Dietel T, Evers C, et al. Expanding Phenotype of de Novo Mutations in GNAO1: Four New Cases and Review of Literature. Neuropediatrics 2017;48(5):371–377.

29. Thiel M, Bamborschke D, Janzarik WG, et al. Genotype-phenotype correlation and treatment effects in young patients with GNAO1 -associated disorders. J. Neurol. Neurosurg. Psychiatry 2023;17(Md):806–815.

30. Waak M, Mohammad SS, Coman D, et al. GNAO1-related movement disorder with life-threatening exacerbations: Movement phenomenology and response to DBS. J. Neurol. Neurosurg. Psychiatry 2018;89(2):220–222.

31. Wirth T, Garone G, Kurian MA, et al. Highlighting the Dystonic Phenotype Related to GNAO1. Mov. Disord. 2022;37(7):1547-1554.

32. Yamamoto EA, Berry M, Harris W, et al. Good Response to Deep Brain Stimulation in Two Forms of Inherited Chorea Related to GNAO1 and Neuroacanthocystosis with Illustrative Videos. Mov. Disord. Clin. Pract. 2022;9(3):401–403.

33. Yamashita Y, Ogawa T, Ogaki K, et al. Neuroimaging evaluation and successful treatment by using directional deep brain stimulation and levodopa in a patient with GNAO1-associated movement disorder: A case report. J. Neurol. Sci. 2020;411(January):2019–2021.

34. Yang X, Niu X, Yang Y, et al. Phenotypes of GNAO1 Variants in a Chinese Cohort. Front. Neurol. 2021;12(May)

35. Yilmaz S, Turhan T, Ceylaner S, et al. Excellent response to deep brain stimulation in a young girl with GNAO1-related progressive choreoathetosis. Child’s Nerv. Syst. 2016;32(9):1567–1568.
