# Supplementary material for: Dyskinetic crisis in GNAO1-related disorders: clinical perspectives and management strategies
Source: Front Neurol. 2024 Jun 6;15:1403815. doi: 10.3389/fneur.2024.1403815 (PMC11188927; doi:10.3389/fneur.2024.1403815)
Supplement: Supplementary file 11 [file Data_Sheet_1.docx]

# Supplemental Data. Material and Methods

## Overview

A Delphi consensus process was conducted, informed by a targeted literature review and clinical expertise. The Delphi panel comprised a steering committee of three nonvoting members (JDOE, JDC, and CR, including the chair of the panel, JDOE) (**Supplemental Data. Table S1**). The steering committee has extensive expertise in *GNAO1*-RD and in the Delphi consensus process. The steering committee selected expert panel members with one or more of the following credentials: they lead a specialized movement disorders clinic; they lead national cohort studies to investigate *GNAO1*-RD; or they are members of the European Reference Network for Rare Neurological Diseases (ERN-RND). In total, 19 experts were invited, and 13 experts accepted the invitation to serve in this Delphi process. Countries represented by this panel included Croatia, France, Germany, Italy, the Netherlands, Turkey, the USA, and the UK. It is essential to mention that the steering committee and these thirteen specialists provide care for over one hundred *GNAO1*-RD patients collectively. After a targeted literature review, the steering committee facilitated several stages: 1) information gathering to obtain expert opinion on topics related to the *GNAO1*-RD dyskinetic crisis; and 2) the generation of a list of draft statements related to the dyskinetic crisis. The Delphi consensus experts voted on these statements to confirm if expert consensus was reached. **Figure 1** provides an overview of the *GNAO1*-RD dyskinetic crisis consensus process.

## Proto-statement Questionnaire

The initial information-gathering stage was performed using a proto-statement questionnaire. The proto-statement questionnaire utilized in this study was developed by the steering committee. The committee identified key areas to be covered, focusing primarily on the description and management of dyskinetic crises in *GNAO1*-RD. These areas encompassed: 1) definition of dyskinetic crisis; 2) characteristic clinical features apart from movement disorders; 3) specific triggers or precipitating factors; 4) differences compared to background movement disorders; 5) distinctive patterns or variations; 6) diagnostic criteria; 7) potential short- and long-term complications; 8) management strategies and interventions; and 9) additional factors and considerations. Further details regarding the questionnaire structure and content can be accessed in **Supplemental Data. Tables.**

## Voting Rounds

The draft statements were voted on over a maximum of 3 rounds (**Figure 2**). As with the proto-statement questionnaire, responses in each round were collected; first round (from July 13, 2023, to August 15, 2023), the second round (from August 29, 2023, to September 11, 2023), and the third round (from September 28, 2023, to October 15, 2023), and were extracted anonymously into an Excel spreadsheet. Participation in at least one of the three rounds was mandatory for each Delphi panel member. In rounds 1, 2, and 3, panel members voted anonymously on their level of agreement with each statement using a 6-point Likert scale (strongly agree, agree, somewhat agree, somewhat disagree, disagree, and strongly disagree). Panel members had the option to provide free-text comments after each section of the survey. Panel members were given no other instructions regarding how they should provide responses.

In all voting rounds, the percentage agreement was compared with a predefined consensus threshold (≥67%) previously used in Delphi processes. Statements that failed to reach the predefined consensus threshold were revised based on the feedback provided by those who disagreed with the statement. Revisions were approved by the steering committee. In round 2, revised statements were voted on in the same way as in the first round. A Zoom meeting on September 13, 2023, with 13 members present, allowed for discussing both approved and disapproved statements. Two new topics emerged for discussion: determining the appropriate terminology for describing this movement disorder phenomenon and exploring medical management strategies for these episodes. Following this, a third round proceeded, involving voting on statements that covered these aspects.

## Literature review.

A thorough search of the MEDLINE database was conducted. The search was performed on May 2, 2023, and updated thereafter on October 16, 2023, and included records of any date. The following search strategy was used: [*GNAO1*]. In addition, any additional relevant studies identified that may have been missed in the initial search were included based on a thorough examination of the full-text articles (**Figure S1**).

## Eligibility criteria

Inclusion criteria for this study included patients of any age with a genetic diagnosis of *GNAO1*-related disorders. Any study design except narrative reviews, conference articles, and editorials was included, and only Spanish and English articles were considered. As for the exclusion criteria, they are as follows:

A. Articles not involving patients or patients with neurological pathology unrelated to *GNAO1*.

B. Articles lacking a detailed description of dyskinetic crises, including at least two of the following characteristics: patient genotype, crisis triggers (or mention of their absence), description of phenomenology (to ensure dyskinetic crises are addressed), management of dyskinetic crises, and description of associated complications.

C. In cases where patients were described in multiple articles, only the article providing the most comprehensive description of the aforementioned characteristics was considered.

D. Exclusion of review articles or caregiver survey-type articles.

E. Articles not written in English or Spanish.

## Study selection

Following the removal of duplicate records, a study selection process was conducted. To ensure consistency, a subset of studies was initially screened to establish a shared understanding of the inclusion and exclusion criteria. JDC and JDOE conducted a trial with the first 10 articles retrieved from the search to ensure uniformity in the criteria for inclusion/exclusion and data extraction. All studies were subsequently assessed based on their titles and abstracts, and those that did not meet the predetermined inclusion criteria were excluded. The full texts of the remaining studies were then reviewed.

## Data extraction

A systematic and standardized approach was undertaken to extract pertinent data from the included studies. A pre-planned and well-designed data extraction sheet was utilized to extract all relevant characteristics of the studies. After conducting a preliminary evaluation of the first ten articles to identify the key characteristics that were consistently relevant and extractable across studies, this methodology was established. The data extraction were performed independently by two reviewers (JDC and JDOE). The extracted data included author, publication year, nomenclature of the motor phenomenon, genotype, MD phenomenology, triggers, management and other comments (including outcome and autonomic symptoms).

## Standard Protocol Approvals, Registrations, and Patient Consents.

Not required for this study. The *GNAO1*-RD patients and parents granted permission to use videos illustrating dyskinetic crises in this publication.
